# Photothrombotic Ischemic Stroke in Rats: A Systematic Review of Experimental Parameters and Therapeutic Applications in Neuroprotection, Neuromodulation, and Post-Stroke Recovery

**DOI:** 10.3390/biomedicines14102177

**Published:** 2026-09-25

**Authors:** Giovanna R. Raci, Fernando A. Oliveira, Nicole M. E. Valle, Olívia F. M. Dias, Arielly H. Alves, Javier B. Mamani, Bruna O. Dall’Ovo, Beatriz A. Tatai, Mariana P. Nucci, Lionel F. Gamarra

**Affiliations:** 1 Hospital Israelita Albert Einstein, São Paulo 05652-000, SP, Brazil; giraci@alumni.usp.br (G.R.R.); fernando.ao@einstein.br (F.A.O.); nicolemev@gmail.com (N.M.E.V.); olivia_fmd@hotmail.com (O.F.M.D.); ariellydahora1997@gmail.com (A.H.A.); javierbm@einstein.br (J.B.M.); brunadallovo@gmail.com (B.O.D.); beatrizakemi.moraes@einstein.edu.br (B.A.T.); 2 LIM44, Hospital das Clínicas, Faculdade de Medicina, Universidade de São Paulo, São Paulo 05403-000, SP, Brazil; mariana.nucci@hc.fm.usp.br

**Keywords:** ischemic stroke, animal behavior, neuroprotection, neuromodulation, post-stroke recovery

## Abstract

**Background/Objectives:** This systematic review analyzed the experimental parameters of the photothrombotic ischemic stroke model in rats and their relationship with neuroprotection, neuromodulation, and post-stroke recovery studies. We examined the follow-up periods and the behavioral, histological, and complementary outcomes employed. **Methods:** The search was conducted in PubMed and Scopus following PRISMA guidelines. Of the 341 records identified, 34 studies met the eligibility criteria. **Results:** Sprague–Dawley, male, and young adult rats predominated, while Rose Bengal was used in all studies. Intravenous administration and transcranial irradiation without craniotomy were the most frequent approaches, with predominantly unilateral lesions targeting motor and sensorimotor regions. Despite this relative uniformity, the considerable variability in irradiation parameters and incomplete reporting of relevant physical characteristics of the protocols were observed, indicating limitations to interlaboratory reproducibility. Post-stroke recovery was the most frequent application, followed by neuromodulation and neuroprotection. Neuroprotection studies were concentrated in earlier time windows and combined a functional assessment with the characterization of lesion, neuronal survival, cell death, oxidative stress, and inflammation. Neuromodulation studies showed greater diversity in stimulation strategies and integrated behavioral outcomes with cellular, neurovascular, perfusion, neuroimaging, and electrophysiological assessments, supporting the investigation of circuit modulation. In contrast, recovery studies employed longer follow-up periods, with greater emphasis on dexterity, asymmetry, and motor performance, associated with assessments of neurogenesis, angiogenesis, neurotrophic factors, and neuronal reorganization. **Conclusions:** Overall, the findings demonstrate that neuroprotection, neuromodulation, and post-stroke recovery require distinct temporal windows and assessment strategies, and that the integrated selection of induction parameters and outcomes should be guided by the therapeutic purpose to improve the consistency and comparability of studies using the photothrombotic model.

## 1. Introduction

Stroke remains one of the leading causes of death and long-term neurological disability worldwide, representing a major challenge for healthcare systems due to its substantial clinical, social, and economic burden. Ischemic stroke, which accounts for approximately 65–70% of all stroke cases, results from the interruption of cerebral blood flow, triggering a complex cascade of pathophysiological events that ultimately leads to progressive brain injury [1,2,3]. According to estimates from the Global Burden of Disease Study 2021, published in 2024, stroke was responsible for approximately 7.3 million deaths and 160.5 million disability-adjusted life years (DALYs), ranking as the third leading cause of death and the fourth leading cause of disability worldwide [1].

Beyond its high mortality rate, stroke profoundly impairs patients quality of life and is associated with persistent neurological sequelae, including motor deficits, spasticity, cognitive impairment, and other long-term functional disabilities [4]. Although reperfusion therapies, including intravenous thrombolysis and mechanical thrombectomy, have expanded the therapeutic options for ischemic stroke, their clinical benefit remains limited to a relatively small proportion of patients due to the narrow therapeutic time window and strict eligibility criteria [5]. As a result, therapeutic strategies targeting post-stroke functional recovery remain limited, highlighting the need for the development of novel therapeutic approaches [6].

Animal models play a crucial role in advancing the understanding of the mechanisms underlying ischemic brain injury and in accelerating the development of novel therapeutic strategies for stroke. The selection of an appropriate experimental model depends on the specific objectives of the study, the therapeutic agent being evaluated, and its mechanism of action [7,8]. Several approaches have been developed to induce experimental stroke in different animal species, with rodents being the most widely used due to their high reproductive capacity, low maintenance costs, availability of transgenic models, and the relative similarity of their cerebrovascular anatomy to that of humans [9,10].

Among rodent species, rats offer several important advantages for ischemic stroke research. Most behavioral tests were originally developed and validated in rats, which exhibit a greater consistency and lower behavioral variability than other commonly used rodent models [11,12]. Furthermore, their larger body size enables more precise surgical procedures, serial blood and cerebrospinal fluid sampling, and the application of neuroimaging and electrophysiological recording techniques, thereby allowing more comprehensive evaluations of brain injury and post-stroke functional recovery [13].

The main experimental models of focal ischemic stroke include middle cerebral artery occlusion (MCAO) using an intraluminal filament, embolic models, endothelin-1-induced ischemia, and the photothrombotic stroke model [14]. Among these, the photothrombotic stroke model is particularly attractive because of its technical simplicity, low surgical mortality, high reproducibility, minimal tissue trauma, and precise control over lesion location and size [9]. This model relies on the local formation of a thrombus through a photochemical reaction triggered by the systemic administration of photosensitive dyes, such as Rose Bengal or Erythrosin B, followed by focal illumination with green light. The photoactivation of the dye induces endothelial injury, platelet activation, and subsequent thrombus formation, leading to local blood flow interruption and the induction of infarction in predefined cortical areas [15,16,17]. Although it only partially recapitulates the ischemic penumbra observed in clinical settings, this model induces consistent motor deficits and enables behavioral, functional, and structural assessments throughout the acute and chronic phases of stroke, making it widely applicable for studies of neural plasticity, functional recovery, and therapeutic intervention evaluation [18,19].

In addition to its extensive application in the investigation of stroke pathophysiology, the photothrombotic model has emerged as an important preclinical platform for evaluating innovative therapeutic strategies [20,21]. Recent studies have utilized this model to investigate neuroprotective strategies aimed at limiting ischemic lesion expansion by reducing neuronal death, modulating inflammatory responses, attenuating oxidative stress, and preserving the blood–brain barrier (BBB) integrity [3,22]. Similarly, neuromodulation strategies, including electrical, magnetic, optical stimulation, and other invasive and non-invasive modalities, have been explored to modulate cortical excitability, promote neural plasticity mechanisms, and facilitate the functional reorganization of neural networks following brain injury [23]. Importantly, this model has been extensively used in studies of post-stroke functional recovery, allowing the investigation of pharmacological, cellular, and gene therapies, biomaterial-based approaches, and motor and cognitive rehabilitation protocols aimed at promoting neurogenesis, angiogenesis, synaptogenesis, and axonal remodeling [24].

In this context, the behavioral assessment represents one of the main experimental endpoints for evaluating the efficacy of these therapeutic interventions [25]. The behavioral tests employed encompass the assessment of coordination and balance, sensorimotor function, and cognitive performance, enabling a comprehensive evaluation of neurological deficits and functional recovery over time [11].

Despite the extensive use of the photothrombotic model, considerable heterogeneity remains among the experimental protocols described in the literature. This variability encompasses animal-related factors, including strain, sex, and age, as well as photosensitizer type and dose, irradiation parameters, lesion location, and behavioral assessment strategies, including the tests employed, timing of evaluations, and outcome measures analyzed [11,25,26]. Such variability hinders comparisons across studies, compromises the reproducibility of findings, and limits the assessment of the translational potential of different therapeutic interventions [3].

Previous reviews have described the main experimental models of ischemic stroke and compared their methodological characteristics, advantages, and limitations, including those of the photothrombotic model [9,17]. More recent methodological work has specifically addressed the principles, applications, limitations, and technical considerations of the photothrombotic stroke model [27]. Collectively, these studies have contributed to the characterization and methodological refinement of photothrombotic stroke models. However, these previous publications have primarily focused on the characteristics of stroke models or on the methodological aspects of photothrombosis, rather than systematically integrating induction parameters with a therapeutic purpose, follow-up periods, and the selection of functional and complementary outcomes. Specifically in rat studies, this relationship has not been systematically examined. An integrated analysis of these aspects may help identify methodological patterns and experimental gaps and improve the comparability and reproducibility of preclinical studies.

Accordingly, this systematic review aims to characterize the experimental parameters used to induce photothrombotic ischemic stroke in rat studies investigating neuroprotection, neuromodulation, and post-stroke recovery through therapeutic interventions and behavioral assessment. In addition to characterizing the induction protocols within this therapeutic and functional context, this review seeks to determine how these distinct therapeutic purposes are related to follow-up periods and the selection of behavioral, histological, and complementary outcomes. This approach enables the identification of methodological patterns specific to each application and provides a basis for selecting protocols and assessment strategies that are better aligned with the experimental purpose.

## 2. Materials and Methods

### 2.1. Search Strategy

This systematic review follows the Preferred Reporting Items for Systematic Reviews and Meta-Analyses (PRISMA) guidelines [28]. We searched for indexed articles, in PubMed and Scopus. The selection criteria were based on the following keyword sequences ((“stroke” OR “ischemic stroke” OR “cerebral ischemia” OR “ischemic cerebrovascular accident”) AND (“photothrombosis” OR “photothrombotic” OR “photochemically” OR “photochemical”) AND (“rat” OR “wistar” OR “sprague-dawley”)) AND (“behavioral assessment” OR “behavior”), and boolean operators were used:

Pubmed: (((((((((Stroke[Title/Abstract]) OR (“Ischemic stroke”[Title/Abstract])) OR (“Cerebral ischemia”[Title/Abstract])) OR (“Ischemic cerebrovascular accident”[Title/Abstract])) AND ((((Photothrombosis[Title/Abstract]) OR (Photothrombotic[Title/Abstract])) OR (Photochemically[Title/Abstract])) OR (Photochemical[Title/Abstract]))) AND (((Rat) OR (Wistar)) OR (“Sprague-Dawley”))) AND ((“Behavioral assessment”) OR (Behavior))))) AND ((“2020”[Date—Publication]: “3000”[Date—Publication]))

Scopus: ((TITLE-ABS-KEY (Stroke) OR TITLE-ABS-KEY (“Ischemic stroke”) OR TITLE-ABS-KEY (“Cerebral ischemia”) OR TITLE-ABS-KEY (“Ischemic cerebrovascular accident”))) AND ((TITLE-ABS-KEY (Photothrombosis) OR TITLE-ABS-KEY (Photothrombotic) OR TITLE-ABS-KEY (Photochemically) OR TITLE-ABS-KEY (Photochemical))) AND ((ALL (Rat) OR ALL (Wistar) OR ALL (“Sprague-Dawley”))) AND ((ALL (“Behavioral assessment”) OR ALL (Behavior))) AND PUBYEAR > 2019 AND PUBYEAR < 2026

This systematic review was prospectively registered in International Prospective Register of Systematic Reviews (PROSPERO) under the identification number CRD420261487486.

### 2.2. Study Eligibility Criteria

This review included exclusively original articles published in English within the last five years. Eligibility criteria were defined based on the PEO strategy, adapted to the review’s objective: Population (P), rats subjected to in vivo ischemic stroke models; Exposure (E), induction of cerebral ischemia via photothrombosis combined with the application of a therapeutic intervention; and Outcome (O), functional assessment of the intervention’s effects using behavioral tests.

Only studies meeting all the following criteria simultaneously were considered eligible: (i) use of rats as an experimental model; (ii) induction of ischemic stroke; (iii) use of photothrombosis as the method for inducing brain injury; (iv) application of a therapeutic intervention aimed such as neuroprotection, neuromodulation, or the promotion of functional recovery post-stroke; and (v) assessment of the intervention’s effects via at least one behavioral test.

Studies meeting one or more of the following conditions were excluded from this review: (i) review articles; (ii) book chapters; (iii) experimental protocols; (iv) letters, communications, or editorials; (v) conference abstracts or proceedings; (vi) publications in languages other than English; (vii) duplicate articles across databases; (viii) studies employing experimental models other than rats; (ix) investigations using ischemic stroke induction methods other than photothrombosis; (x) studies lacking a therapeutic intervention; and (xi) studies that did not perform behavioral assessments or that relied exclusively on global neurological assessment scales without applying specific behavioral tests.

Therefore, studies exclusively focused on methodological characterization of the photothrombotic model, without a therapeutic intervention and behavioral outcome assessment, were outside the predefined scope of this review.

### 2.3. Data Collection

Study selection was conducted by six reviewers (G.R.R., F.A.O., N.M.E.V., A.H.A., J.B.M., and M.P.N.), randomly organized into pairs, who independently examined the titles and abstracts of all publications identified in the PubMed and Scopus databases, following a pre-defined search strategy. Publications deemed potentially relevant were retrieved in full for comprehensive reading and eligibility analysis by the same reviewers. In cases of disagreement, decisions were discussed with an independent reviewer (L.F.G.), who was responsible for evaluating inconsistencies and ensuring the consistent application of selection criteria; the final inclusion of studies was determined by consensus among all reviewers.

### 2.4. Data Extraction

Data from the included studies were extracted in a standardized manner, considering the characteristics of the animals used (strain, sex, weight, and age); the characteristics of the photosensitizer (agent, dose, concentration, and route of administration); and aspects related to the preparation of the ischemic model (performance of craniotomy, lesion site, and the interval between photosensitizer administration and irradiation).

Irradiation parameters were also extracted, including the light source, power, application time, and the distance between the light source and the target. To evaluate the model, information was collected regarding the study objective, experimental groups, follow-up period, behavioral tests employed, and frequency of assessments, as well as histological analyses, complementary evaluations, and the main reported outcomes.

Data identification and extraction were performed by the authors. Subsequently, the data were reviewed, consolidated, and organized into two summary tables: one covering aspects of inducing the ischemic stroke model via photothrombosis, and the other covering aspects of evaluating the photothrombotic model.

### 2.5. Risk of Bias Assessment

Risk of bias was assessed using the Systematic Review Centre for Laboratory Animal Experimentation (SYRCLE) Risk of Bias tool [29], a tool specifically developed to evaluate methodological quality and potential sources of bias in animal intervention studies. Based on the Cochrane Risk of Bias framework, the SYRCLE tool assesses ten methodological domains covering selection bias (sequence generation, baseline characteristics, and allocation concealment), performance bias (random housing and blinding of caregivers and investigators), detection bias (random outcome assessment and blinding of outcome assessors), attrition bias (incomplete outcome data), reporting bias (selective outcome reporting), and other potential sources of bias. Each domain was judged according to the SYRCLE guidelines and classified as low risk, high risk, or unclear risk, depending on the information reported by each study [29]. The SYRCLE assessment was independently performed by three reviewers (G.R.R.; N.M.E.V.; and A.H.A.). Disagreements were resolved through discussion until consensus was reached.

To measure inter-rater agreement, the Fleiss’ kappa coefficient was calculated, which indicates the level of concordance among multiple raters, with values ranging from 0 (no agreement) to 1 (perfect agreement) [30]. Calculations were carried out using JASP software (v0.98.1; https://jasp-stats.org/; accessed on 27 June 2026). Graphical summaries of the risk-of-bias assessments were generated using the robvis R package (version 0.3.0.900) and its associated Shiny web application [31].

### 2.6. Data Analysis

The results were analyzed descriptively and presented using absolute frequencies and percentage distributions for the variables extracted from the tables, enabling comparisons among the included studies and with the available scientific literature. For quantitative variables, the respective ranges were also considered to highlight the breadth of the reported methodological and experimental parameters.

## 3. Results

### 3.1. Selection Process of the Articles Identified According to the PRISMA Guidelines

**Figure 1:**
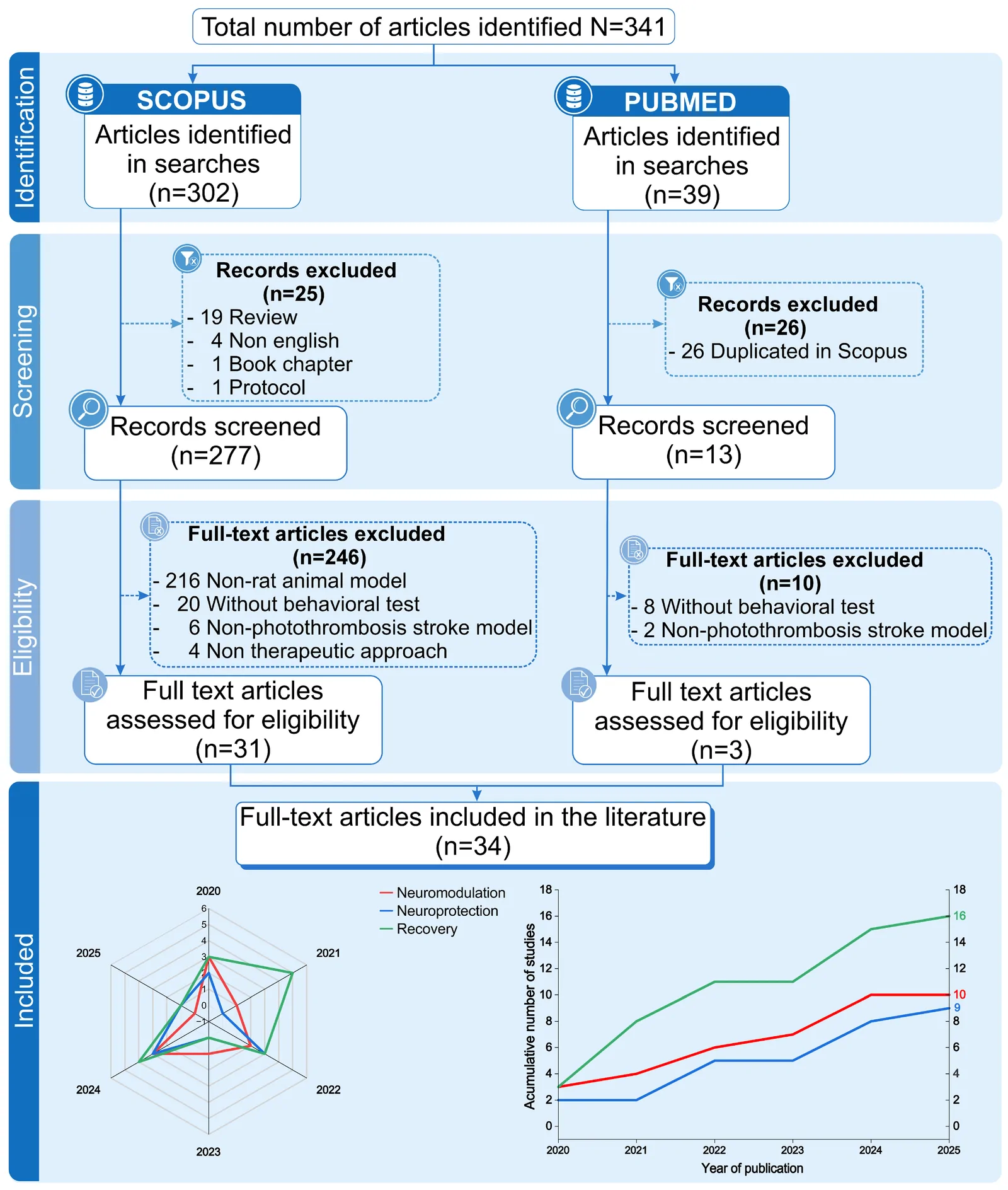
PRISMA flow diagram illustrating the study selection process (upper panel). The radar chart (lower-left panel) presents the annual distribution of the included studies according to their therapeutic application: neuromodulation (red), neuroprotection (blue), and post-stroke recovery (green). The line graph (lower-right panel) shows the cumulative number of studies published over time for each therapeutic application, using the same color coding.

Searches were conducted for publications indexed in the PubMed and Scopus databases, considering studies published within the last five years. A total of 341 articles were identified, including 302 from Scopus and 39 from PubMed. In the Scopus database, after excluding 19 review articles, 4 non-English articles, 1 book chapter, and 1 experimental protocol, 277 studies remained for screening. Following full-text assessment, 246 articles were excluded, including 216 that used non-rat animal models, 20 that did not include behavioral tests, 6 that employed non-photothrombotic stroke models, and 4 that did not evaluate therapeutic interventions, resulting in the inclusion of 31 studies. In the PubMed database, 26 duplicate articles identified in Scopus were removed, leaving 13 studies for screening. After full-text assessment, 10 articles were excluded, including 8 that did not include behavioral tests and 2 that employed non-photothrombotic stroke models, resulting in the inclusion of 3 studies. Overall, the systematic selection process resulted in 34 studies that met all predefined eligibility criteria and were included in the qualitative synthesis [32,33,34,35,36,37,38,39,40,41,42,43,44,45,46,47,48,49,50,51,52,53,54,55,56,57,58,59,60,61,62,63,64,65], comprising 31 studies identified through Scopus and 3 additional studies identified through PubMed. The complete study selection process is summarized in the PRISMA flow diagram presented in the upper section of Figure 1.

The included studies were distributed throughout the analyzed period, with the highest number of publications observed in 2024 (29.4%; *n* = 10) [34,35,36,37,38,39,40,41,42,43], followed by 2020 (23.5%; *n* = 8) [58,59,60,61,62,63,64,65], 2022 (20.6%; *n* = 7) [45,46,47,48,49,50,51], 2021 (17.6%; *n* = 6) [52,53,54,55,56,57], 2025 (5.9%; *n* = 2) [32,33], and 2023 (2.9%; *n* = 1) [44]. Thus, although studies were identified throughout the evaluated period, their annual distribution was heterogeneous, with a marked concentration of publications in 2024. Regarding therapeutic objectives, functional recovery emerged as the predominant research focus, comprising 16 studies (47.1%) [33,34,38,39,41,45,47,48,52,53,54,55,56,61,63,64], followed by neuromodulation with 10 studies (29.4%) [35,36,37,44,46,50,57,59,60,65] and neuroprotection with 9 studies (26.5%) [32,40,42,43,46,49,51,58,62]. These therapeutic categories were not necessarily mutually exclusive, as some studies investigated mechanisms or outcomes encompassing more than one therapeutic domain, as observed in study [46], which was classified under both neuromodulation and neuroprotection. The annual distribution of these therapeutic applications is illustrated in the radar chart presented in the lower-left section of Figure 1.

The temporal distribution of therapeutic applications showed distinct patterns across the analyzed period (lower-right section of Figure 1). Functional recovery exhibited the greatest cumulative increase, from 3 studies in 2020 to 16 studies by 2025. Neuromodulation increased from 3 studies in 2020 to 10 studies by 2024, with no additional studies identified in 2025, whereas neuroprotection increased from 2 studies in 2020 to 9 studies by 2025. Overall, functional recovery represented the predominant therapeutic application of the rat photothrombotic stroke model, followed by neuromodulation and neuroprotection.

### 3.2. Results of the Risk of Bias Assessment

**Figure 2:**
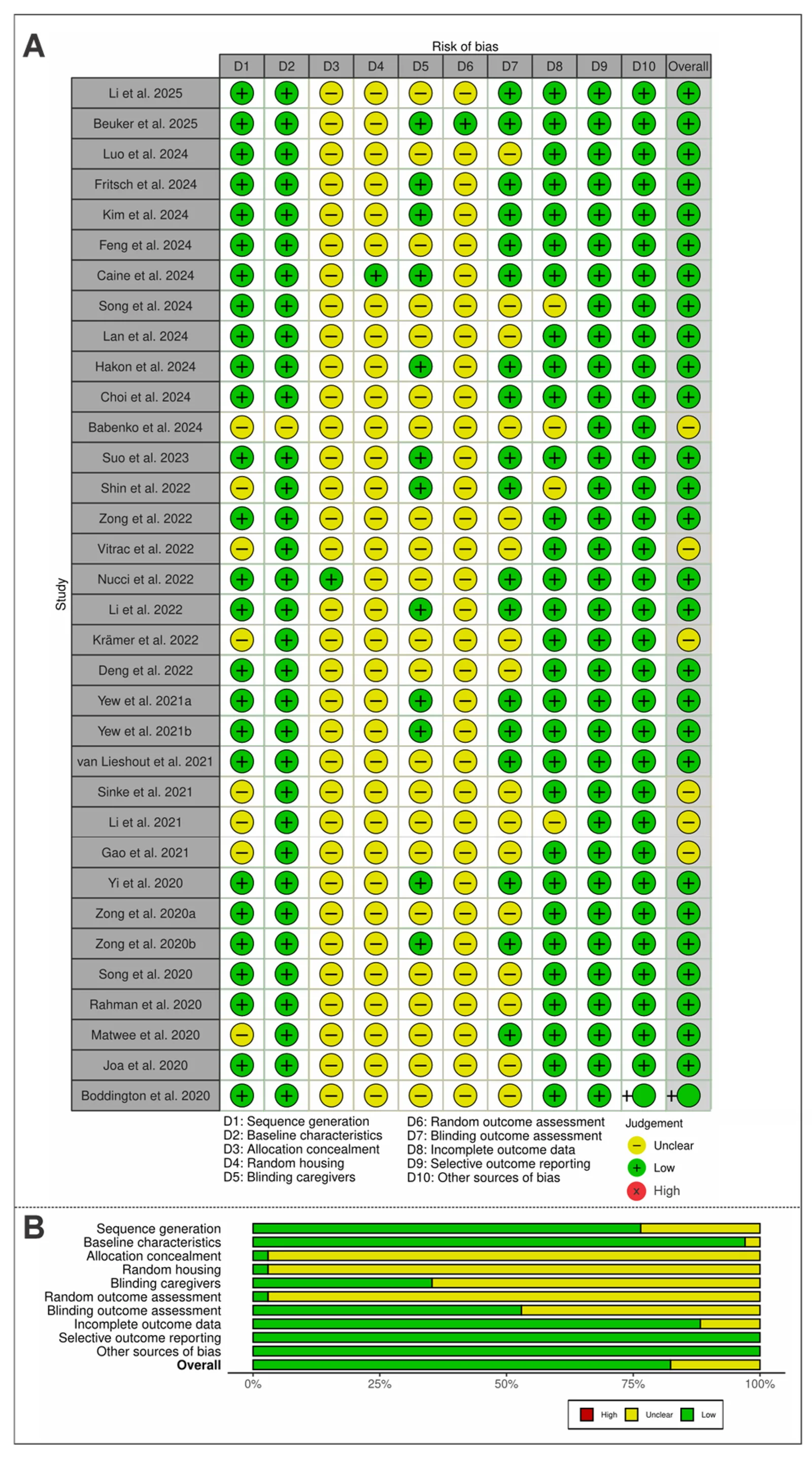
Risk of bias assessment of the included studies using the SYRCLE Risk of Bias tool. (**A**) Study-level risk-of-bias judgments for the 34 included studies [32,33,34,35,36,37,38,39,40,41,42,43,44,45,46,47,48,49,50,51,52,53,54,55,56,57,58,59,60,61,62,63,64,65] across the ten domains (D1 to D10) and the overall assessment. Each cell indicates the assigned risk level based on consensus among reviewers: green (+) for low risk and yellow (−) for unclear risk. The “Overall” column reflects the overall risk of bias rating across all ten domains. (**B**) Summary of the distribution of risk-of-bias judgments across the ten domains, expressed as the percentage of studies classified as low or unclear risk.

The methodological quality of the 34 studies included in this review was assessed using the SYRCLE Risk of Bias tool, which evaluates ten methodological domains related to potential sources of bias in animal intervention studies (Figure 2). Overall, most domains were classified as low or unclear risk of bias, reflecting adequate reporting of some methodological aspects while highlighting insufficient methodological information in several studies.

Regarding sequence generation (D1) and baseline characteristics (D2), most studies were classified as low risk of bias. However, allocation concealment (D3) was predominantly judged as unclear risk, as information regarding group allocation procedures was rarely reported. The domains related to performance bias, including random housing (D4) and blinding of caregivers (D5), were predominantly classified as unclear risk, reflecting the limited reporting of these methodological procedures. Similarly, random outcome assessment (D6) was frequently judged as unclear risk, whereas blinding of outcome assessment (D7) was predominantly classified as low risk of bias. Incomplete outcome data (D8), selective outcome reporting (D9), and other sources of bias (D10) were predominantly classified as low risk of bias, indicating that these methodological aspects were generally well reported. Overall, the assessment indicated generally low risk in several domains; however, methodological reporting was insufficient for allocation concealment, random housing, caregiver blinding, and random outcome assessment, resulting in frequent unclear-risk judgments for these domains.

To assess the reliability of these judgments, Fleiss’ Kappa was calculated to determine inter-rater agreement across the three reviewers. The overall agreement was considerable (κ = 0.679, standard error = 0.099; 95% CI: 0.458–0.846).

### 3.3. Animal Characteristics

**Figure 3:**
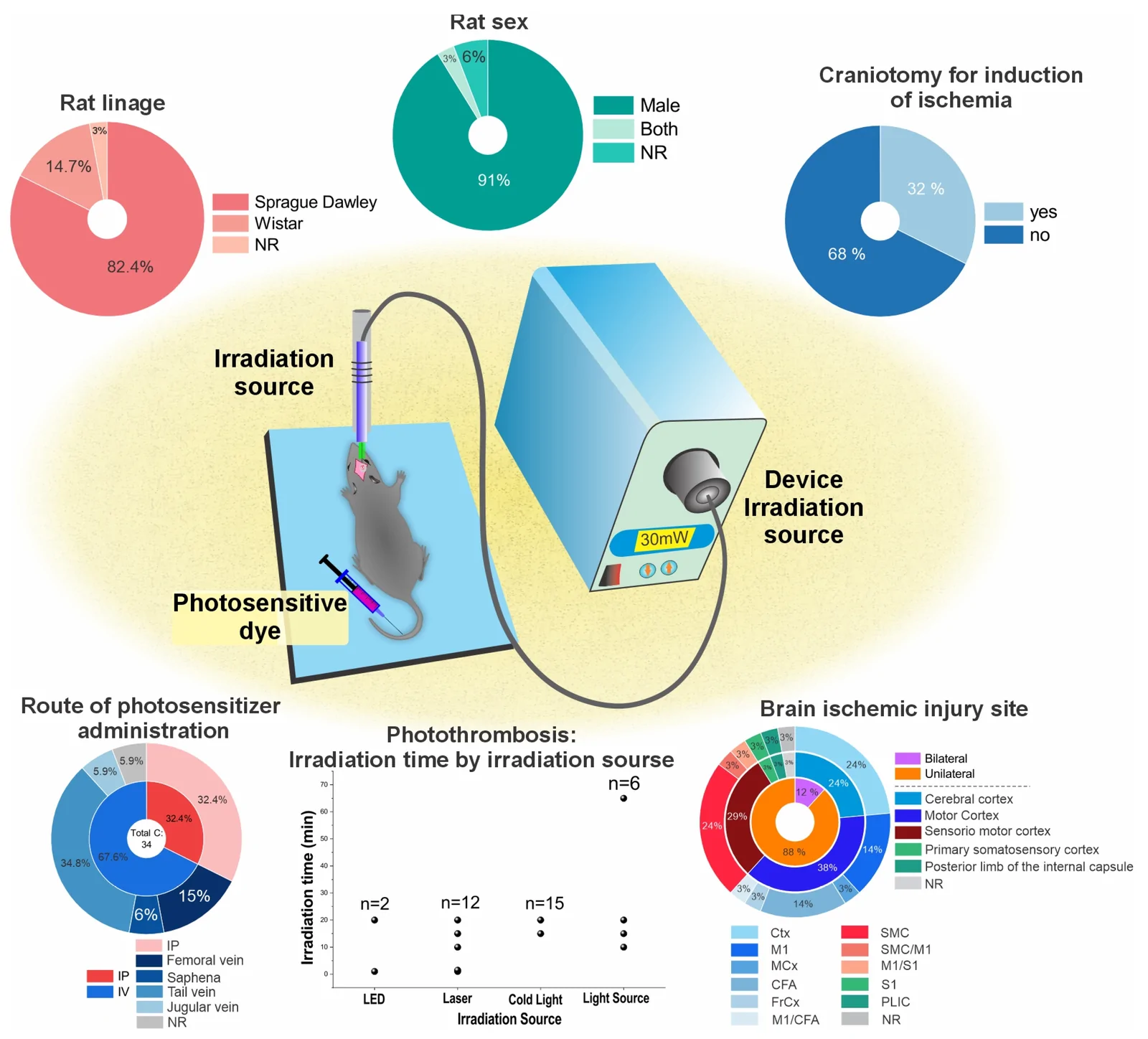
Overview of the main experimental characteristics of the photothrombotic ischemic stroke models included in the systematic review. The central schematic illustrates the general procedure for photothrombotic stroke induction, comprising systemic administration of a photosensitive dye followed by focal brain irradiation. The surrounding graphs summarize the distribution of rat strain, sex, use of craniotomy, route of photosensitizer administration, irradiation source and exposure time, and anatomical site and laterality of the ischemic lesion across the included studies. Irradiation sources included LED, laser, cold light, and other light sources, with substantial variability in exposure duration among protocols. Brain injury was predominantly induced in cortical motor and sensorimotor regions. IP, intraperitoneal; IV, intravenous; Ctx, cortex; M1/MCx, motor cortex; SMC, sensorimotor cortex; S1, primary somatosensory cortex; CFA, caudal forelimb area; FrCx, frontal cortex; PLIC, posterior limb of the internal capsule; NR, not reported.

The included studies were analyzed according to the animal characteristics used in photothrombotic stroke protocols, including strain, sex, age, and body weight. Sprague-Dawley rats predominated, being used in 28 of the 34 studies (82.4%) [32,34,35,36,37,38,39,40,41,44,45,46,47,49,51,52,53,54,55,56,57,58,59,60,61,62,63,64], whereas Wistar rats were used in five studies (14.7%) [33,42,48,50,65]. One study (2.9%) did not report the rat strain [43], as shown in Table 1 and Figure 3.

Regarding sex, 31 studies (91.2%) exclusively used male rats [32,33,34,35,36,37,38,39,40,41,42,44,46,47,48,49,50,51,52,53,54,55,56,57,59,60,61,62,63,64,65], whereas one study included animals of both sexes [58], and two studies (5.9%) did not report sex [43,45]. Regarding body weight, 15 studies (44.1%) used rats weighing between 250 and 350 g [32,34,36,42,44,48,50,52,53,54,56,57,61,62,64]. Three articles (8.8%) used animals weighing less than 250 g [40,51,59], whereas two studies (5.9%) used animals weighing more than 350 g [35,55]. Body weight was not reported in 14 studies (41.2%) [33,37,38,39,41,43,45,46,47,49,58,60,63,65].

Regarding age, 23 studies (67.6%) used rats predominantly within the 8–12-week age range [32,33,34,35,36,37,39,40,42,44,46,47,48,50,51,54,55,56,60,61,62,63,64]. Three studies (8.8%) used animals older than 12 weeks [33,38,49], whereas one study (2.9%) reported a younger age range of 6–9 weeks [65]. Two studies (5.9%) specifically employed neonatal rats [45,58], while five studies (14.7%) did not report animal age [41,43,52,53,59].

Overall, the experimental evidence was strongly concentrated on young adult male Sprague-Dawley rats, particularly animals aged approximately 8–12 weeks.

The anesthetic protocols used during the induction of photothrombotic stroke in the included studies were analyzed according to the therapeutic purpose. Isoflurane was the most frequently used anesthetic, reported in 20 studies (58.8%) [34,35,37,38,39,40,41,44,45,47,48,49,50,51,54,55,58,61,62,63]. A combination of ketamine and xylazine was selected in six studies (17.6%) [33,36,42,52,53,64], followed by sodium pentobarbital in four (11.8%) [32,46,59,60] and propofol in two (5.9%) [56,57]. Chloral hydrate [43] and a combination of ketamine and medetomidine [65] were reported in one study each (2.9%). Among the neuromodulation studies, isoflurane was used in four of the ten studies (40.0%) [35,37,44,50] and sodium pentobarbital in three (30.0%) [46,59,60]. Combinations of ketamine and xylazine [36] and of ketamine and medetomidine [65], as well as propofol [57], were used in one study each (10.0%). In the neuroprotection studies, isoflurane predominated (5/9; 55.6%) [40,49,51,58,62], followed by sodium pentobarbital (2/9; 22.2%) [32,46]. A combination of ketamine and xylazine [42] and chloral hydrate [43] were used in one study each (11.1%). Among the post-stroke recovery studies, isoflurane was used in 11 of the 16 studies (68.8%) [34,38,39,41,45,47,48,54,55,61,63], the combination of ketamine and xylazine in four (25.0%) [33,52,53,64] and propofol in one (6.3%) [56]. The choice of anesthetic represents an important methodological consideration, particularly in studies evaluating neuroprotection, because anesthetic agents may modulate cerebral physiology and ischemic injury and may therefore influence the observed therapeutic response. In addition, the interval between anesthesia and behavioral assessment may affect functional outcomes because of potential residual anesthetic effects. However, information on post-anesthetic recovery time was inconsistently reported across the included studies, precluding a systematic comparison of this parameter. Therefore, heterogeneity in anesthetic protocols and incomplete reporting of recovery time should be considered potential sources of variability when interpreting therapeutic and functional outcomes.

Regarding the drugs used for euthanasia, the combination of ketamine and xylazine was used in five studies (14.7%) [33,35,42,52,53], followed by isoflurane in three (8.8%) (8.8%) [38,51,63], and sodium pentobarbital [47,59], and chloral hydrate [43,56] in two studies each (5.9%), while ketamine alone [36], and urethane [65] were selected in one study each (2.9%). In neuromodulation studies, the combination of ketamine and xylazine [35], ketamine [36], sodium pentobarbital [59], and urethane [65] were used in one study each (10.0%). In neuroprotection studies, the combination of ketamine and xylazine [42], chloral hydrate [43], and isoflurane [51] were used in one study each (11.1%). Among post-stroke recovery studies, the combination of ketamine and xylazine predominated, appearing in three studies (18.8%) [33,52,53], followed by isoflurane in two (12.5%) [38,63] and sodium pentobarbital [47] and chloral hydrate [56] in one study each (6.3%).

Euthanasia was generally performed at predefined experimental endpoints according to the follow-up period and tissue-collection schedule of each study. However, specific humane endpoint criteria for early euthanasia, such as excessive weight loss, severe neurological deterioration, inability to feed or drink, or persistent signs of distress, were not consistently reported across the included studies. Consequently, a systematic comparison of humane endpoint criteria according to the different observation periods was not possible. This incomplete reporting represents an additional methodological limitation and highlights the importance of clearly describing both scheduled euthanasia and predefined humane endpoints in future studies.

### 3.4. Photothrombotic Stroke Induction

The analysis of experimental protocols revealed that Rose Bengal was the only photosensitizer used in the studies included in this review (100%) [32,33,34,35,36,37,38,39,40,41,42,43,44,45,46,47,48,49,50,51,52,53,54,55,56,57,58,59,60,61,62,63,64,65]. Despite this uniformity in photosensitizer selection, considerable methodological variability was observed in how Rose Bengal was administered and subsequently activated to induce the ischemic lesion. This variability involved the administration route, dose and solution concentration, preparation of the irradiation site, anatomical target, and timing between photosensitizer administration and light exposure (Table 1 and Figure 3).

Among the 34 included studies, 23 (67.6%) administered Rose Bengal intravenously [33,34,35,36,38,39,40,41,43,44,47,50,51,52,53,54,55,56,57,61,62,63,65], indicating that intravenous delivery was the predominant approach for photothrombotic stroke induction. Among these studies, doses between 10 and 20 mg/kg were the most frequently used (7/23; 30.4%) [36,38,47,52,53,62,63], and three of these studies reported solution concentrations ranging from 10 to 40 mg/mL [47,52,53]. A second group of studies employed substantially higher doses (>40 mg/kg; 8/23, 34.8%) [34,39,40,44,51,56,57,61], with reported concentrations ranging from 15 to 20 mg/mL when available. Three studies (13.0%) used doses below 10 mg/kg [33,35,65] of which only one (4.3%) reported the solution concentration (10 mg/mL) [33]. Intermediate dose ranges were less frequently represented: one study used 20–30 mg/kg [55], whereas another used 30–40 mg/kg with a concentration of 30 mg/mL [43].

The choice of venous access also varied, although the lateral tail vein clearly predominated, being used in 12 studies (34.8% of all included studies) [34,35,36,38,40,44,47,51,56,57,62,63]. The femoral vein was used in 14.7% of studies [33,39,52,53,61], whereas the jugular [43,65] and saphenous veins [54,55] were each used in 5.9% of studies. In two studies (5.9%), the specific vein used for administration was not specified [41,50].

In contrast, intraperitoneal administration represented a less frequently adopted but still substantial methodological approach, being used in 11 studies (32.4%) [32,37,42,45,46,48,49,58,59,60,64]. Within this group, the dosing protocols showed two predominant patterns: five studies (45.5%) used doses ranging from 10 to 25 mg/kg [42,45,49,58,64], whereas another five studies used 100 mg/kg [32,37,46,59,60]. One study (9.1%) reported only the Rose Bengal solution concentration (50 mg/mL), without specifying the corresponding dose [48]. Thus, although the same photosensitizer was used throughout the included literature, the route and amount administered were far from standardized.

Following photosensitizer administration, a second major source of methodological variation concerned how the target brain region was prepared for illumination. Eleven studies (32.4%) performed a craniotomy to expose the cerebral cortex [34,36,40,44,47,48,51,56,57,58,65]. However, transcranial illumination through the intact skull was substantially more common, being used in 21 studies (61.8%) [32,33,35,37,39,41,42,43,45,46,49,50,52,53,54,55,59,60,61,62,64]. Two additional studies (5.9%) adopted an intermediate approach in which the skull was preserved but thinned before irradiation [38,63]. Overall, these findings show that nearly two-thirds of the studies favored induction without craniotomy, whereas direct cortical exposure was used in approximately one-third of the protocols.

The anatomical distribution of the lesions further demonstrated that the model was primarily directed toward cortical regions associated with motor and sensorimotor function. The motor cortex, including regions described as primary motor cortex (M1), motor cortex (MCx), caudal forelimb area (CFA), and frontal cortex (FrCx), was the most frequently targeted site, being reported in 13 studies (38.2%) [33,35,38,40,42,47,50,51,52,53,57,63,65]. The sensorimotor cortex (SMC, SMC/M1, and M1/S1) accounted for another 10 studies (29.4%) [34,39,41,44,54,55,58,61,62,64]. Together, these two categories represented more than two-thirds of the included studies, demonstrating the strong predominance of lesions involving motor-related cortical territories. Eight studies (23.5%) induced lesions in cortical regions reported more generally as cortex (Ctx) [32,37,43,45,46,56,59,60]. The primary somatosensory cortex (S1) [48] and the posterior limb of the internal capsule (PLIC) [36] were each targeted in one study, while one study (2.9%) did not report the lesion site [49].

A similarly consistent pattern was observed for lesion laterality. Most studies induced unilateral lesions (88.2%) [33,34,35,36,38,39,40,41,42,44,45,47,48,49,50,51,52,53,54,55,56,57,58,59,60,61,62,63,64,65], whereas only four studies (11.8%) induced bilateral cortical lesions [32,37,43,46]. Therefore, the predominant experimental configuration consisted of a unilateral cortical lesion involving motor or sensorimotor territories.

Finally, the interval between Rose Bengal administration and irradiation showed a clear relationship with the administration route. Intravenous protocols generally used short circulation intervals before light exposure. In six studies (17.6%), irradiation was initiated immediately after intravenous injection of the dye [38,39,47,52,53,55], while another six initiated irradiations within the first 2 min [33,34,35,56,57,65]. Only one intravenous study reported a longer interval of 10 min [41]. By contrast, intraperitoneal administration was consistently associated with longer intervals before irradiation, with five studies using a 5-min interval [37,42,46,59,60], one using 10 min [49], one 20 min [48], two 30 min [45,58], and one 40 min [32]. This distribution indicates that the timing of photoactivation was closely associated with the selected administration route, with intravenous protocols typically proceeding to irradiation almost immediately and intraperitoneal protocols allowing a longer interval before light exposure.

### 3.5. Irradiation Parameters

Irradiation parameters are critical determinants of the location, extent, and reproducibility of photothrombotic lesions. In this section, the type of light source, equipment power, application time, and distance between the source and the target tissue were analyzed, as presented in Table 1 and Figure 3.

White or cold light was the most frequently employed source, being used in 15 studies (44.1%) [35,37,40,41,42,46,47,49,50,54,55,59,60,62,65]. Laser irradiation was used in 12 studies (34.8%) [32,33,34,36,38,43,44,51,56,57,58,63], whereas light-emitting diode based (LED-based) sources were described in two studies (5.9%) [32,45]. In six studies (17.6%), the authors used generic terms without clearly characterizing the technology responsible for light generation [39,48,52,53,61,64]. Notably, Li et al. [32] employed both LED and laser irradiation in the same protocol. Some studies also reported additional characteristics, including wavelength, optical filters, beam diameter, or the use of optical fibers for light delivery [32,33,34,36,37,38,41,42,43,44,45,46,48,49,50,51,52,53,55,56,57,58,59,60,63,64].

Regarding power, 17 studies (50.0%) reported neither the nominal power of the equipment nor the power effectively applied to the tissue [32,33,37,40,41,42,45,47,48,49,50,51,54,55,59,60,64]. Seven studies (20.6%) reported the use of equipment with a nominal power of 150 W, although the power effectively delivered to the target was not specified [35,39,46,52,53,61,62]. Among the studies that used lasers and reported the applied power, five (14.7%) used values below 35 mW [34,36,56,57,63], whereas three (8.8%) used values ranging from 35 to 100 mW [38,43,44]. Yi et al. [58] reported a nominal power of 36 mW but did not specify the power effectively applied. Boddington et al. [65] used a cold-light source with a nominal power ranging from 4 to 5 W. The studies that used LED irradiation did not report the irradiation power [32,45].

Application time ranged from 1 to 65 min. A duration of 15 min was the most frequent, being used in 13 studies (38.2%) [34,37,43,44,46,49,50,52,53,56,57,59,60]. Exposure for 20 min was described in 11 studies (32.4%) [32,33,35,41,42,47,54,55,62,64,65]. A duration of 10 min was used in four studies (11.8%) [38,39,61,63], whereas exposure periods longer than 20 min were reported in three studies (8.8%) [40,48,51]. Another three studies (8.8%) used exposure times shorter than 2 min [36,45,58].

The distance between the light source and the target was the least frequently reported parameter. In 27 studies (79.4%), this information was not specified [33,34,35,37,38,39,40,41,42,43,44,45,47,49,50,51,54,55,56,57,58,59,60,61,62,63,65]. In three studies (8.8%), the source was positioned as close as possible to the skull without direct contact [52,53,64]. Li et al. [32] positioned the light source 1 mm above the skull, whereas Kim et al. [36] inserted the optical fiber to a depth of 7.8 mm toward the posterior limb of the internal capsule. Nucci et al. [48] positioned the source 5 mm from the dura mater, and Zong et al. [46] maintained the light source in direct contact with the skull.

### 3.6. Study Purpose

Following the characterization of the experimental parameters used for photothrombotic stroke induction, the included studies were grouped according to the main therapeutic application of the model into three categories: neuroprotection, neuromodulation, and post-stroke recovery. Neuroprotection included studies primarily aimed at evaluating the preservation of neural tissue or the attenuation of ischemic injury and its associated pathological processes. Neuromodulation comprised studies designed to investigate interventions intended to modulate neural activity, excitability, or plasticity in the context of stroke. Post-stroke recovery included studies focused on the restoration or improvement of behavioral, motor, sensorimotor, cognitive, or neurological function following stroke. This classification was used in subsequent analyses to compare behavioral, histological, molecular, and other experimental outcomes across the three therapeutic applications (Table 2).

Post-stroke recovery was the most frequently investigated application, comprising 16 studies (47.1%) [33,34,38,39,41,45,47,48,52,53,54,55,56,61,63,64]. Neuromodulation was investigated in 10 studies (29.4%) [35,36,37,44,46,50,57,59,60,65], whereas neuroprotection was investigated in nine studies (26.5%) [32,40,42,43,46,49,51,58,62]. The categories were not mutually exclusive when a study addressed more than one therapeutic application. Accordingly, study [46] addressed both neuromodulatory and neuroprotective effects and was included in both categories; therefore, the category-specific percentages do not sum to 100% (Table 2).

### 3.7. Follow-Up

Follow-up duration after photothrombotic stroke induction varied considerably across studies and showed distinct temporal patterns according to the main experimental application. Among the 16 studies investigating functional recovery, follow-up was predominantly oriented toward longer-term assessments. Two studies (12.5%) evaluated animals within 24 h [45,48] and only one (6.3%) performed assessments between >24 h and 7 days [52]. Two studies (12.5%) reported follow-up periods of >7 to 14 days [41,47]. In contrast, the most frequently represented follow-up periods were >14 to 28 days [33,48,53,54,64] and >28 days [38,39,52,56,63] each reported in five studies (31.3%). Three studies (18.8%) did not report the follow-up period [34,55,61] (Table 2).

Neuromodulation studies showed a comparatively greater concentration of assessments within the first week after stroke induction. None of the 10 studies evaluated animals within the first 24 h. Follow-up between >24 and 72 h was reported in three studies (30%) [35,44,50]. whereas >3 to 7 days was the most frequently represented interval, occurring in five studies (50%) [37,46,50,59,60], Four studies (40%) extended follow-up to >14–28 days [36,46,59,60], while only one study included assessments beyond 28 days [46], One study did not report the follow-up period [57], and another performed follow-up after completion of all electrophysiological experiments [65].

In contrast to functional recovery studies, neuroprotection studies were more strongly represented at early post-stroke time points. Among the nine studies investigating neuroprotective effects, four (44.4%) performed follow-up within 24 h [32,40,51,58], whereas none reported assessments between >24 and 72 h. Four studies (44.4%) evaluated animals between >3 and 7 days [43,46,58,62], two (22.2%) between >7 and 14 days [32,42], and one (11.1%) between >14 and 28 days [46]. Follow-up beyond 28 days was reported in two studies (22.2%) [32,46]. In addition, one study (11.1%) defined follow-up according to euthanasia after completion of behavioral testing [49].

Overall, the distribution of follow-up periods differed according to the experimental purpose of the studies. Functional recovery investigations were characterized by longer observation periods, with most assessments extending beyond 14 days, whereas neuromodulation studies were more concentrated within the first week and neuroprotection studies showed a greater representation of very early assessments, particularly within the first 24 h. These findings indicate substantial heterogeneity in follow-up duration across the included studies, while also revealing application-specific temporal patterns in outcome assessment.

### 3.8. Assessments

#### 3.8.1. Behavioral Assessment

Motor function was the most frequently evaluated behavioral domain, being assessed in 91.2% of the included studies [32,33,34,35,36,37,38,40,41,42,43,44,45,46,47,48,49,50,51,52,53,54,55,56,58,59,60,62,63,64,65]. Within this domain, the behavioral assessment profile was predominantly directed toward forelimb function and skilled motor performance rather than global neurological impairment. Fine motor dexterity was investigated in 58.8% of the studies [33,34,35,36,37,41,43,46,47,50,52,53,54,56,59,62,64], primarily using the Single Pellet Reaching Test (SPRT) (23.5%) [36,47,50,52,53,54,56,64], the Adhesive Removal Test (ART) (17.6%) [33,37,41,47,59,62], the Forelimb Placing Test (FPT) (14.7%) [34,43,46,52,53], and Pasta handling task (2.9%) [35]. Forelimb use asymmetry constituted another recurrent component of motor assessment, with the Cylinder Test being used in 32.4% of studies [33,34,37,38,42,43,48,53,55,62,63] (Table 2).

Other dimensions of motor performance were evaluated less frequently and were distributed across tests targeting locomotor coordination, strength, endurance, balance, and exploratory activity. Locomotion on narrow or uneven surfaces was assessed in 26.5% of the studies using Beam walking test (BWT) (14.7%) [32,37,41,46,49], Ladder rung walking test (LRWT) (8.8%) [37,44,59], and Grip-walking task (2.9%) [65]. Strength and endurance tests were evaluated in 20.6% of the studies using the Wire Hanging Test (WHT) (11.8%) [37,45,58,59] and the Grip Strength Test (8.8%) [41,49,60]. By comparison, assessments of spontaneous locomotion, dynamic balance, postural responses, and global neurological deficits were relatively uncommon. The Open Field Test (OFT) was used in 11.8% of studies [32,40,48,51], the Rotarod Test in 8.8% [33,62,64], and the Treadmill Test in 2.9% [32]. Postural coordination and reflexes were assessed using the Postural Hang Reflex (PHR) (5.9%) [41,60] or Paw Placement Test (PPT) (2.9%) [35], whereas sensorimotor asymmetry was evaluated using the Corner Test in one study (2.9%) [62]. Global neurological scales were used only as complementary measures in 8.8% of studies, including the modified Neurological Severity Score (mNSS) (5.9%) [35,44] and the Neuroscore (2.9%) [33].

The predominance of motor assessment was consistent across the three therapeutic applications, encompassing all neuroprotection studies [32,40,42,43,46,49,51,58,62], 90.0% of neuromodulation studies [35,36,37,44,46,50,59,60,65], and 87.5% of post-stroke recovery [33,34,38,41,45,47,48,52,53,54,55,56,63,64]. Thus, despite differences in therapeutic purpose, motor performance constituted the common behavioral endpoint across the included literature, whereas the specific tests used to characterize motor impairment and recovery were considerably more heterogeneous.

**Figure 4:**
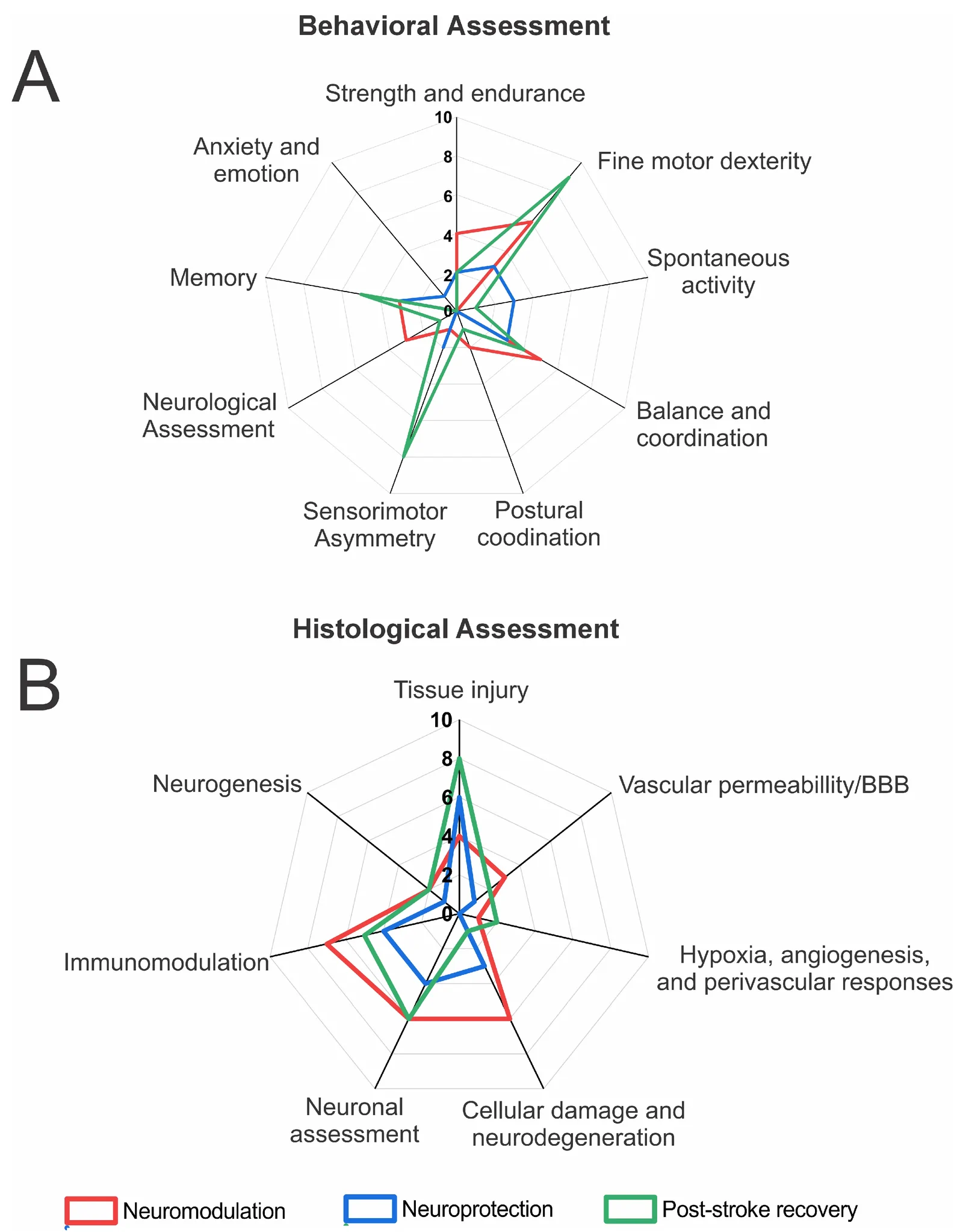
Distribution of behavioral and histological assessments according to the therapeutic application of the photothrombotic stroke model. Radar plots show the number of studies assessing each type of assessment across neuromodulation (red), neuroprotection (blue), and post-stroke recovery (green). (**A**) Behavioral assessment. (**B**) Histological assessment. Values represent the number of studies within each therapeutic application that assessed the corresponding assessment.

In contrast to the extensive assessment of motor outcomes, cognitive-related behavioral domains were investigated in less than one-third of the studies. Memory function was assessed in 29.4% [32,33,38,39,42,46,57,59,61,64], with spatial memory representing the most frequently investigated cognitive component (17.6%), primarily through the Morris Water Maze (MWM) (11.8%) [32,33,39,61] and Radial Maze Task (RMT) (5.9%) [59,64]. Recognition memory was assessed using the Novel Object Recognition (NOR) test in only one study (2.9%) [46]. Reward-based motor learning was evaluated using the Staircase Test (5.9%) [38,42], whereas instrumental learning and motivation were investigated using the Lever Press Task (2.9%) [57]. Despite its lower overall representation, memory assessment was proportionally similar across therapeutic applications, occurring in 30.0% of neuromodulation studies [46,57,59], 31.3% of post-stroke recovery studies [33,38,39,61,64], and 33.3% of neuroprotection studies [32,42,46]. Behavioral domains beyond motor and cognitive function were rarely explored: only one neuroprotection study [32] assessed anxiety using the Elevated Plus Maze (EPM) and affective behavior using the Sucrose Preference Test (SPT). The distribution of behavioral assessments according to therapeutic application is summarized in Figure 4A. Behavioral outcomes were predominantly concentrated on motor and sensorimotor domains, with distinct profiles across neuroprotection, neuromodulation, and post-stroke recovery studies.

The temporal distribution of behavioral testing revealed an additional pattern across the included studies, with assessments predominantly extending beyond the acute post-stroke phase. Only three studies performed behavioral testing within the first 24 h after stroke induction [40,45,51], while 10 studies included assessments within the first 7 days [33,35,37,42,43,44,49,50,58,62], and five extended testing to 2 weeks [34,41,42,47,59]. In contrast, half of the included studies (*n* = 17; 50.0%) conducted behavioral assessments beyond 2 weeks [32,33,36,38,39,46,48,52,53,54,55,56,57,60,61,63,64], with follow-up extending to 60 days in some protocols. Taken together, the behavioral evidence was characterized by three main features: a strong predominance of motor outcomes, substantial heterogeneity in the specific tests used to capture motor function, and a greater emphasis on medium and long-term behavioral assessment than on acute testing.

#### 3.8.2. Histological Assessment

Histological assessments were used to characterize a functional continuum of post-ischemic responses, from structural tissue injury and neurovascular alterations to neuronal damage, glial responses, and mechanisms of repair and plasticity (Table 2). Structural tissue characterization was the most frequent endpoint, reported in 19 studies (55.9%; *n* = 19/34) [32,33,38,40,41,42,44,45,47,48,50,51,52,58,59,60,62,63,65]. Nissl-based staining, particularly cresyl violet, together with 2,3,5-Triphenyl tetrazolium chloride (TTC) and hematoxylin and eosin (H&E), was mainly used to define infarct extent and tissue architecture, while Golgi-Cox staining provided additional information on neuronal and dendritic morphology [34]. Tissue injury assessment was more frequent in neuroprotection studies (66.7%; *n* = 6/9) [32,40,42,51,58,62] and recovery studies (50.0%; *n* = 8/16) [33,38,41,45,47,48,52,63] than in neuromodulation studies (30.0%; *n* = 3/10) [44,59,65].

Neurovascular characterization was less frequent and combined markers of endothelial structure, BBB integrity, permeability, perfusion, hypoxia, and vascular remodeling. Vascular and BBB endpoints were evaluated in five studies (14.7%) [33,34,37,44,60], using CD31, rat endothelial cell antigen-1 (RECA-1), glucose transporter 1 (GLUT1), zonula occludens-1 (ZO-1), claudin-5, and occludin, whereas permeability and perfusion were assessed in four studies (11.8%) [33,37,44,60] using Evans Blue, IgG extravasation, and FITC-dextran. Hypoxia and vascular remodeling were investigated in three studies (8.8%) [52,53,60] using markers including hypoxia-inducible factor 1-alpha (HIF-1α), vascular endothelial growth factor (VEGF), Collagen IV, and platelet-derived growth factor receptor beta (PDGFRβ). Thus, neurovascular assessment extended beyond vascular structure to functional BBB disruption and peri-infarct remodeling, but remained less represented than structural lesion analysis.

At the cellular level, neuronal injury and preservation were characterized using complementary markers of apoptosis, degeneration, and neuronal survival. Cell death and DNA damage were assessed in seven studies (20.6%) [37,44,45,46,59,60,62], mainly using TUNEL, cleaved caspase-3, phosphorylated H2A.X, and Fluoro-Jade C, whereas NeuN was used to characterize mature neuronal populations in 12 studies (35.3%) [33,36,40,41,44,45,46,51,52,53,58,60]. Glial responses were also prominent (38.2%; *n* = 13/34) [34,35,37,44,45,46,49,52,53,58,59,60,62], primarily through glial fibrillary acidic protein (GFAP) and ionized calcium-binding adaptor molecule 1 (Iba1), with C3d and S100 calcium-binding protein A10 (S100A10) providing additional characterization of reactive astrocytic phenotypes [59,60]. Inflammatory and immunomodulatory pathways were less frequently investigated (11.8%; *n* = 4/34) [44,49,59,60], using interleukin-1 beta (IL-1β), interleukin-10 and its receptor (IL-10/IL-10R), transforming growth factor beta (TGF-β), and nuclear factor kappa B (NF-κB).

Mechanisms of endogenous repair were comparatively less represented. Neurotrophic signaling was evaluated in four studies (11.8%; *n* = 4/34) [36,46,61,62], mainly through brain-derived neurotrophic factor/ tropomyosin receptor kinase B (BDNF/TrkB), whereas neurogenesis and cell proliferation were assessed in four studies (11.8%) [33,46,52,60]. These markers covered different stages of tissue repair, including neural progenitor populations identified by nestin and SRY-box transcription factor 2 (SOX2), immature neurons identified by doublecortin (DCX), neural lineage differentiation assessed using class III β-tubulin (Tuj1) and oligodendrocyte transcription factor 2 (Olig2), and proliferative activity evaluated using 5-bromo-2′-deoxyuridine (BrdU), Ki-67, 5-chloro-2′-deoxyuridine (CldU), and 5-iodo-2′-deoxyuridine (IdU). Synaptic and dendritic plasticity was evaluated in three studies (8.8%). [53,59,60], whereas neuronal subtype characterization was reported in two studies (5.9%) [36,41]. Oxidative stress was among the least frequently assessed mechanisms (5.9%; *n* = 2/34) [58,59].

Overall, histological assessment was primarily centered on structural tissue injury, glial responses, and neuronal survival, whereas neurovascular, inflammatory, neurogenic, synaptic, and oxidative mechanisms were less consistently investigated. The assessment profile also varied according to therapeutic objective: neuroprotection studies emphasized tissue injury and neuronal preservation, neuromodulation studies incorporated a broader cellular and neurovascular characterization, whereas functional recovery studies showed less consistent tissue-level assessment. The distribution of histological assessment domains is shown in Figure 4B, encompassing tissue injury, neuronal assessment, immunomodulation, vascular-related responses, and neurogenesis, with different relative patterns among the three therapeutic applications.

#### 3.8.3. Complementary Assessments

Complementary assessments varied across the included studies and reflected differences in the biological questions addressed by the photothrombotic stroke model. Biochemical and molecular analyses were the most frequent complementary approaches, being reported in 14 studies (41.2%; *n* = 14/34) [37,39,40,42,44,45,46,49,51,52,59,60,62,64]. Nine studies (26.5%; *n* = 9/34) [32,34,36,43,48,50,54,55,56] relied exclusively on functional or structural imaging, two studies (5.9%; *n* = 2/34) [57,65] focused on electrophysiological or neural-signal measurements, and two studies (5.9%; *n* = 2/34) [61,63] performed general biochemical analyses. Seven studies (20.6%; *n* = 7/34) did not report complementary assessments [33,35,38,41,47,53,58] (Table 2).

The distribution of complementary assessments varied according to therapeutic objective. Neuroprotection studies predominantly incorporated biochemical and molecular analyses [40,42,46,49,51,62]. Neuromodulation studies also frequently included molecular biology assessments [37,44,46,59,60], but showed a broader integration of imaging [36,37,50,59,60] and electrophysiological assessments [57,65]. Functional recovery studies, in turn, combined biochemical and molecular analyses [39,45,52,64] with functional and structural imaging [34,48,54,55,56].

The biological material used for these analyses consisted predominantly of brain tissue, including ischemic, peri-infarct, and ipsilateral cortical regions, as well as the ipsilesional hippocampus [37,39,40,42,44,45,46,49,51,52,59,60,62,64]. Serum was additionally analyzed in three studies [37,49,51], and isolated cerebral microvessels were used in one study [37]. Thus, assessments of cell death and neuroinflammation were primarily tissue-based, whereas circulating biochemical markers were evaluated less frequently. Biochemical and molecular assessments were performed at multiple time points across the included studies, ranging from 2 h to 35 days after photothrombotic stroke induction, covering the hyperacute, acute, subacute, and chronic phases of the post-stroke response. Hyperacute assessments (2–24 h) were reported in three studies [40,45,51], acute assessments (3–7 days) [37,44,46,49,52,59,62], subacute assessments (13–22 days) [42,46,59,60], and chronic assessments (29–35 days) [46,52]. In two studies [39,64], biochemical and molecular analyses were performed after a 4-week treadmill exercise intervention. Thus, biochemical and molecular assessments encompassed multiple phases of the post-stroke response, with sampling schedules determined by the specific biological processes and therapeutic interventions investigated.

Among the biochemical and molecular approaches, western blotting was the most frequently used method (38.2%; *n* = 13/34) [37,39,40,42,45,46,49,51,52,59,60,62,64], followed by ELISA [37,40,49], nitric oxide/nitric oxide synthase (NO/NOS) measurements [40,51], qPCR [42,45], RT-qPCR [44,49], NADPH oxidase activity [59], and cytokine arrays [59,60]. These techniques targeted distinct biological processes, with assessments at specific time points across the post-ischemic response: oxidative stress and antioxidant status (ROS, MDA, SOD, GSH, NO/NOS, and NADPH oxidase) [40,51,59], assessed from 2 h to 21 days after stroke; neurotrophic and intracellular signaling (BDNF, TrkB, FGF2, PI3K/Akt, NF-κB, SIRT1, PPARγ, and AMPK/mTOR) [39,40,42,46,49,62,64], assessed from 24 h to 35 days; blood–brain barrier integrity and vascular remodeling (ZO-1, claudin-5, occludin, collagen IV, PDGFRβ, VEGF, and HIF-1α) [37,44,52,60], assessed from 3 to 29 days; and tissue repair and neurogenesis (DCX, nestin, SOX2, Tuj1, Olig2, MBP, BrdU, and Ki67) [42,46,52,60], assessed from 3 to 35 days. Among these, cell death and neuroinflammation were the most frequently investigated processes. These assessments were predominantly performed in brain tissue collected from ischemic, peri-infarct, or ipsilateral regions at different stages after stroke induction. For cell death, the markers assessed included TUNEL, caspase-3 (cleaved form and enzymatic activity), caspase-9, Bax and Bcl-2, Fluoro-Jade C, and JNK/phospho-JNK/PARP-1 [37,40,44,45,46,51,59,60,62], evaluated from 2 h to 35 days after stroke. For neuroinflammation, the markers assessed included GFAP [37,44,45,46,49,52,60,62] and Iba1 [37,45,46,52,62], inflammatory cytokines (IL-1β, TNF-α, IL-6, IL-10, and TGF-β) [40,44,45,49,59,60], microglial polarization markers (CD68, CD206, CD32, CD74, CD86, and ARG1) [45,52,59], and signaling proteins (NF-κB and MMP-2/MMP-9) [44,49,60], evaluated from 24 h to 35 days after stroke.

Magnetic resonance imaging (MRI) was the most frequently reported imaging modality (20.6%; *n* = 7/34) [34,43,49,50,54,55,56], followed by positron emission tomography/computed tomography (PET/CT) (8.8%; *n* = 3/34) [34,36,50]. Other approaches, including laser speckle contrast imaging (LSCI) and laser doppler flowmetry (LDF), were used to characterize cerebral perfusion and hemodynamic changes. Electrophysiological assessments were less frequently used [57,65].

Overall, complementary assessments showed distinct methodological profiles across the three therapeutic applications, with biochemical and molecular approaches more strongly represented in neuroprotection, and imaging, perfusion, and electrophysiological approaches more broadly incorporated in neuromodulation. Functional recovery studies showed less consistent use of complementary assessments.

### 3.9. Outcomes

Of the 34 included studies, 16 investigated post-stroke recovery (during the acute, subacute, or chronic phases) [33,34,38,39,41,45,47,48,52,53,54,55,56,61,63,64], 10 evaluated neuromodulation approaches [35,36,37,44,46,50,57,59,60,65], and 9 investigated neuroprotective strategies [32,40,42,43,46,49,51,58,62]. These therapeutic categories were not mutually exclusive, as study [46] addressed both neuromodulation and neuroprotection (Table 2).

Regarding post-stroke recovery, the investigated approaches comprised exercise-based, pharmacological, cellular, nutritional, and mechanistic strategies. Exercise was the most recurrent therapeutic approach, with beneficial recovery-related outcomes reported in five studies [38,39,48,61,64], including combinations with cell therapy [48] or nutritional intervention and environmental enrichment [38]. Cell therapy and nutritional interventions were also investigated independently [53,63]. Pharmacological approaches were evaluated in five studies, including ghrelin [33], safinamide [34], metabotropic glutamate receptor 5 negative allosteric modulator (mGluR5-NAM) [41], poly(lactic-co-glycolic acid) PLGA nanoparticles loaded with perampanel [45], and olomoucine [52]. Four studies did not apply therapeutic interventions but instead investigated determinants of post-stroke recovery, including dopamine transmission [47], translational aspects of recovery [54], degeneration in regions adjacent to the lesion [56], and the contribution of specific brain structures to neural communication and functional recovery [55].

Neuromodulation studies encompassed electrical, magnetic, optical, and circuit-based approaches applied at different post-stroke phases. These included transcranial direct current stimulation (tDCS) [35], optogenetic modulation or photostimulation [36,44], photobiomodulation (PBMT) [37], continuous theta-burst stimulation (cTBS) [46], subthalamic nucleus high-frequency stimulation (STN-HFS) [50], modulation of high-frequency local field potential activity [57], repetitive transcranial magnetic stimulation (rTMS) [59,60], and intermittent theta-burst stimulation [65]. Collectively, these approaches were investigated across acute, subacute, and chronic stages of stroke recovery.

Neuroprotection studies predominantly investigated pharmacological and molecular interventions. Beneficial effects were reported following Salvianolic Acid A [32], γ-aminobutyric acid type A receptor (GABAAR) receptor activation [40], Neuropeptide FF [42], lithium ions [43], leonurine combined with L-nitroargininemethyl ester (L-NAME) [51], combined Aspirin (ASA), clopidogrel, and Coenzyme Q10 (CoQ10) treatment [58], and anti-pro brain-derived neurotrophic factor (BDNF) intervention [62]. In addition, one study examined the influence of maternal high-fat diet on astrocytic characteristics and stroke outcomes [49].

Overall, distinct intervention profiles were observed across the three therapeutic domains: functional recovery studies encompassed rehabilitative, pharmacological, cellular, and mechanistic approaches; neuromodulation studies showed the greatest diversity of stimulation modalities and intervention timing; and neuroprotection studies were predominantly centered on pharmacological and molecular strategies.

## 4. Discussion

The present systematic review aimed to synthesize the protocols employed over the last five years for inducing the photothrombotic model of ischemic stroke in rats, considering its applications in neuroprotection, neuromodulation, and post-stroke recovery, as well as the methods used to assess their functional effects. Overall, the studies showed considerable homogeneity in baseline aspects of the experimental design, particularly regarding animal strain and the photosensitizing agent used, reinforcing the consolidation of the photothrombotic model, originally described by Watson et al. in 1985 [15], as a widely used model for investigating the pathophysiological mechanisms of stroke and testing therapeutic interventions. However, this standardization did not extend equally to lesion induction parameters, follow-up periods, and assessment methods, with differences observed in photosensitizer dose and route of administration, irradiation characteristics, lesion location, follow-up duration, and selection of behavioral tests [32,33,34,35,36,37,38,39,40,41,42,43,44,45,46,47,48,49,50,51,52,53,54,55,56,57,58,59,60,61,62,63,64,65].

In addition to this methodological heterogeneity, the synthesis of the 34 studies revealed that experimental design and the outcomes employed varied according to the therapeutic purpose investigated. Post-stroke recovery was the most frequent application (47.1%; 16/34) [33,34,38,39,41,45,47,48,52,53,54,55,56,61,63,64], followed by neuromodulation (29.4%; 10/34) [35,36,37,44,46,50,57,59,60,65] and neuroprotection (26.5%; 9/34) [32,40,42,43,46,49,51,58,62]. These differences involved not only the type of intervention but also the temporal window of assessment, the nature of the behavioral tests, histological characterization, and the use of molecular, imaging, and electrophysiological analyses [32,33,34,35,36,37,38,39,40,41,42,43,44,45,46,47,48,49,50,51,52,53,54,55,56,57,58,59,60,61,62,63,64,65].

This relationship between therapeutic purpose and experimental design is particularly relevant in the photothrombotic model, whose properties may represent advantages or limitations depending on the research question. The high spatial precision and relative reproducibility of the lesion favor studies requiring associations among anatomical location, functional deficits, and neural circuits, whereas the photochemical mechanism of microvascular occlusion and the characteristics of peri-infarct tissue should be considered in investigations dependent on penumbra and reperfusion [66,67,68]. Consequently, the suitability of the model should be assessed according to the experimental purpose, since neuroprotection, neuromodulation, and post-stroke recovery address distinct biological processes and require different temporal windows and assessment strategies.

### 4.1. Experimental Characteristics and Induction Parameters of the Photothrombotic Stroke Model

The included studies showed relative homogeneity regarding animal characteristics, with a predominance of young adult, male, Sprague-Dawley rats, whereas greater variability was observed in the parameters directly related to photothrombotic induction [32,33,34,35,36,37,38,39,40,41,42,43,44,45,46,47,48,49,50,51,52,53,54,55,56,57,58,59,60,61,62,63,64,65]. Sprague-Dawley and Wistar rats are among the most commonly used strains in experimental research [69,70]. However, strain selection may influence experimental outcomes, including infarct volume and cerebral edema. Genetic and anatomical differences among strains, particularly variations in pial collateral circulation, may further contribute to variability in the extent of ischemic injury. Although Sprague-Dawley rats are widely used in preclinical stroke research, greater variability in infarct volume has been reported in this strain [71,72].

Although mice are also extensively used in photothrombotic stroke research, the present review was restricted to rats to provide a species-specific synthesis and reduce the additional methodological variability associated with interspecies differences. The use of rats in photothrombotic stroke models offers practical advantages for some experimental applications compared with mice, as their relatively larger brains facilitate stereotaxic procedures, electrophysiological recordings, and neuroimaging, while their broad behavioral repertoire enables the assessment of multiple components of sensorimotor function [68,73]. These characteristics are particularly relevant to the scope of the present review, which emphasizes the relationship between experimental parameters, therapeutic applications, and functional outcomes. Thus, restricting the analysis to rats enabled a more consistent comparison of induction protocols, therapeutic approaches, and outcome-assessment strategies within a single species.

In contrast, the induction parameters showed greater heterogeneity, including photosensitizer dose and route of administration, skull preparation, brain region, light source, power, and irradiation duration [32,33,34,35,36,37,38,39,40,41,42,43,44,45,46,47,48,49,50,51,52,53,54,55,56,57,58,59,60,61,62,63,64,65]. In addition, some physical parameters were incompletely reported, particularly the distance between the light source and the target tissue, which was absent in 79.4% of the studies. This limitation is relevant because lesion extent depends on the interaction between photosensitizer availability and the light energy effectively delivered to the tissue. Therefore, protocol variability, together with incomplete reporting of irradiation parameters, may limit interlaboratory reproducibility and comparison of intervention efficacy [67,68].

However, the relative homogeneity of the included animals also represents an important translational limitation. In this review, 91.2% of the studies [32,33,34,35,36,37,38,40,41,42,43,44,45,46,47,48,49,50,51,52,53,54,55,56,58,62,63,64,65] used exclusively male rats, with a predominance of young adult animals and limited representation of aged animals or models incorporating clinically relevant comorbidities. This experimental profile only partially reflects the heterogeneity of the human stroke population, as sex, age, and comorbidities such as hypertension, diabetes, obesity, and hypercholesterolemia may influence ischemic pathophysiology, lesion evolution, and responses to therapeutic interventions [74]. Translational recommendations, including those proposed by the Stroke Therapy Academic Industry Roundtable (STAIR)) [75], emphasize that therapeutic strategies showing efficacy in young, healthy animals should subsequently be evaluated in both sexes, aged animals, and models incorporating clinically relevant comorbidities. Therefore, although the predominance of young adult male Sprague-Dawley rats may reduce some sources of biological variability across studies, it also limits external validity and the generalizability of the findings to the clinical stroke population, highlighting the need for greater biological diversity in future preclinical studies [76].

These induction characteristics also distinguish photothrombosis from other models of focal cerebral ischemia. Photoactivation of Rose Bengal promotes endothelial damage, platelet aggregation, and localized microvascular thrombosis, providing spatial control and relative reproducibility of the lesion [66,67,68]. In contrast, MCAO produces ischemia over a broader vascular territory and allows the investigation of reperfusion, although lesion extent is more dependent on vascular anatomy, collateral circulation, and occlusion duration [66,68]. Thromboembolic models more directly reproduce thrombotic occlusion and recanalization, but generally present greater variability in lesion location and extent [67]. Thus, the main experimental advantage of photothrombosis lies the spatial control and reproducibility of the induced lesion, whereas its suitability depends on whether these characteristics are compatible with the biological process being investigated.

This consideration becomes particularly important in relation to the ischemic penumbra. While MCAO produces a more evident gradient between the core and potentially salvageable hypoperfused tissue, conventional photothrombosis generally presents a more abrupt transition between infarcted and preserved tissue, with a more limited classical penumbra [66,67]. However, this characteristic is not invariable, since modifications of the photothrombotic protocol can produce regions of perfusion–diffusion mismatch compatible with potentially salvageable tissue [77]. This difference is particularly relevant considering the profiles identified in this review: neuroprotection studies were more focused on early assessments and tissue preservation [32,40,42,43,46,49,51,58,62], whereas neuromodulation studies incorporated assessments of neural and physiological responses [35,36,37,44,46,50,57,59,60,65], and recovery studies showed a higher frequency of prolonged follow-up periods and functional outcomes [33,34,38,39,41,45,47,48,52,53,54,55,56,61,63,64].

Anesthetic protocols represent another relevant source of methodological variability in photothrombotic stroke studies. Isoflurane was the predominant anesthetic in the included studies (58.8%), whereas ketamine/xylazine, sodium pentobarbital, propofol, chloral hydrate, and ketamine/medetomidine were less frequently used. This variability is particularly relevant when interpreting neuroprotective outcomes, as anesthetic agents can modulate cerebral blood flow, metabolism, neurovascular coupling, and other processes involved in ischemic injury [78]. Moreover, isoflurane, the most frequently used anesthetic in the included studies, has demonstrated neuroprotective effects in experimental cerebral ischemia through mechanisms involving oxidative stress, neuroinflammation, blood–brain barrier integrity, and cell-death pathways [79]. Post-anesthetic recovery time was inconsistently reported across the included studies, limiting assessment of whether residual anesthetic effects could have influenced subsequent behavioral evaluations. Therefore, both anesthetic choice and recovery time should be more consistently reported and considered when comparing therapeutic and functional outcomes across photothrombotic stroke studies.

### 4.2. Neuroprotection, Tissue Preservation, and Functional Outcomes

The nine studies classified as neuroprotection [32,40,42,43,46,49,51,58,62] presented an experimental profile predominantly directed toward the early limitation of ischemic damage and the preservation of tissue and function. This orientation was reflected in the follow-up: 44.4% performed assessments within the first 24 h [32,40,51,58], while assessments between 3 and 7 days were also frequent [43,46,58,62]. This temporal pattern is consistent with the principle of neuroprotection, which seeks to interfere with mechanisms secondary to ischemia, including excitotoxicity, mitochondrial dysfunction, oxidative stress, neuroinflammation, and cell death, before the consolidation of irreversible damage [80,81].

The observed histological profile reinforces this mechanistic orientation. Structural characterization of the lesion was performed in 66.7% of the studies [32,40,42,51,58,62], and preservation of mature neurons in 33.3% [40,51,58], complemented by assessments of cell death [62], oxidative stress [58], glial responses [49,58,62], and inflammatory responses [49]. Thus, histology allowed the assessment of different levels of the neuroprotective response, from the extent of tissue damage to cellular mechanisms associated with neuronal survival and the post-ischemic microenvironment. This approach is consistent with external photothrombotic studies in which histological, inflammatory, and metabolic changes were related to tissue preservation and functional response [82,83,84,85]. In the study by Ahmed et al. [85], for example, the reduction in infarct volume was accompanied by increased peri-infarct neurogenesis, reduced gliosis and inflammation, and improvement in neurological deficits, illustrating the importance of integrating cellular mechanisms and function.

However, histological preservation does not necessarily imply functional benefit, making it particularly relevant that motor function was assessed in all neuroprotection studies [32,40,42,43,46,49,51,58,62]. The tests employed covered different sensorimotor components, including locomotor activity using the Open Field [32,40,51], coordination and locomotion using the Beam Walking [32,46,49], limb asymmetry using the Cylinder Test [42,43,62], forelimb function using Forelimb Placing [43,46], strength and endurance [49,58], and coordination using the Rotarod [62]. These tests are not functionally equivalent and may exhibit different sensitivities according to lesion location and the timing of assessment [86,87]. Furthermore, the relationship between infarct volume and behavioral deficits may differ according to the experimental model: in a direct comparison between MCAO and photothrombosis, the association between infarct volume and neurobehavioral alterations was less consistent in the photothrombotic model [88]. This finding reinforces that reductions in lesion size or cell death should be interpreted together with behavioral outcomes capable of demonstrating whether the preserved neural substrate supports neurologically relevant function.

The translational interpretation of these results should also consider the predominance of young adult animals and early therapeutic windows. Age, comorbidities, lesion variability, and timing of treatment initiation may modify neuroprotective efficacy and contribute to the discrepancy between preclinical and clinical results [89,90]. The Stroke Therapy Academic Industry Roundtable (STAIR) recommendations also emphasize the need to reproducibly establish dose–response relationships and therapeutic windows, integrate histological and functional outcomes, and subsequently extend validation to more biologically representative models [75]. Thus, the reproducibility of the photothrombotic lesion favors mechanistic investigations of neuroprotection, but the evidence becomes biologically and translationally more robust when it demonstrates convergence between modulation of the injury mechanism, preservation of the neural substrate, and maintenance of function.

### 4.3. Neuromodulation and Neural Responses

The ten studies classified as neuromodulation [35,36,37,44,46,50,57,59,60,65] employed different stimulation modalities but converged toward a common purpose: to modify the activity, excitability, or plasticity of neural circuits remaining after the lesion. This profile was also reflected temporally: no study restricted follow-up to the first 24 h; 30% assessed periods between >24 and 72 h [35,44,50], and 50% included assessments between >3 and 7 days [37,46,50,59,60], with protocols extending into the subacute and chronic phases [36,46,59,60]. This distribution is biologically consistent with the temporal evolution of excitability and network reorganization after stroke, indicating that the stimulation window should be interpreted in relation to the neural process intended to be modulated rather than solely as a source of methodological heterogeneity [91,92].

The observed histological profile was more diverse than that identified for neuroprotection and was less exclusively focused on tissue preservation. Glial responses were assessed in 60% of the studies [35,37,44,46,59,60], cell death/apoptosis in 50% [37,44,46,59,60], and neuronal survival in 40% [36,44,46,60], in addition to vascular, BBB, and perfusion alterations [37,44,60]. This diversity is consistent with the fact that reorganization after a focal lesion involves not only neurons but also changes in the peri-infarct microenvironment, connectivity, and plasticity of surviving networks [73]. Some included studies also incorporated electrophysiology, local field potential (LFP), neuroimaging, or hemodynamic measures [36,37,44,50,57,59,60,65], allowing cellular and tissue alterations to be more closely related to measures directly associated with the physiological response to neuromodulation, rather than inferring its mechanism solely from behavior.

This distinction is particularly important when interpreting behavioral outcomes. Motor function was assessed in most studies [35,36,37,44,46,50,59,60,65], including measures of dexterity and forelimb function such as SPRT, ART, Forelimb Placing, and Pasta Handling [35,36,37,46,50,59,60], as well as coordination and locomotor tests [37,44,59,65]. These tests assess distinct components of sensorimotor function and exhibit different sensitivities throughout post-stroke progression [86,87]. In particular, more specific tests of dexterity or sensorimotor integration may reveal alterations not detected by global measures, whereas spontaneous recovery or the development of compensatory strategies may reduce the sensitivity of certain tests over time [87,93]. Therefore, in neuromodulation studies, the choice of behavioral test should be functionally related to the stimulated circuit and the assessment window.

Behavioral improvement alone, however, does not demonstrate that the target circuit was effectively modulated. Integrating behavioral outcomes with electrophysiological, neuroimaging, perfusion, or cellular measures [36,37,44,50,57,59,60,65] provides stronger support for target engagement, allowing a more consistent relationship to be established between stimulation, neural/physiological modification, and functional response. This approach is particularly relevant because behavioral performance and tissue damage may follow distinct temporal trajectories after stroke [93], and the effects of neuromodulation depend on the target, stimulation parameters, and functional state of the network [91,92].

Thus, neuromodulation studies showed an assessment profile distinct from that observed in neuroprotection: whereas in the latter histology was predominantly oriented toward demonstrating tissue preservation, in neuromodulation the combination of cellular, physiological, and behavioral outcomes allows investigation of whether the intervention effectively modifies surviving circuits and whether this modification translates into functional benefit. The anatomical precision of the photothrombotic lesion favors this type of investigation, although the structural and functional heterogeneity of human stroke remains an important consideration for translation [73,91].

### 4.4. Post-Stroke Recovery and Functional Outcomes

Post-stroke recovery represented the most frequent application of the photothrombotic model in this review (47.1%; 16/34) [33,34,38,39,41,45,47,48,52,53,54,55,56,61,63,64] and showed the longest temporal profile. Follow-up periods of >14–28 days [33,48,53,54,64] and >28 days [38,39,52,56,63] were the most frequent (31.3% each), whereas assessments restricted to the first 24 h were poorly represented [45,48]. This pattern is consistent with the need to monitor not only the evolution of deficits but also processes of motor learning, plasticity, and neural reorganization that develop during the subacute and chronic phases [24,94].

The behavioral profile observed was consistent with this longitudinal purpose, with tests targeting different components of motor recovery. Fine motor dexterity was investigated using tests such as the Single Pellet Reaching Test (SPRT) and Adhesive Removal Test (ART) [33,41,47,52,53,54,56,64], whereas asymmetry in limb use was frequently monitored using the Cylinder Test [33,34,38,48,53,55,63], complemented by assessments of coordination, strength, and motor performance [33,38,41,45,47,52,53,54,55,56,63,64]. These outcomes are not functionally equivalent: improvement in a specific task may reflect restitution of the impaired function, task-dependent learning, or the development of compensatory strategies [24,87,95,96]. This distinction is illustrated by the included studies themselves, since rehabilitation-induced muscular changes were not necessarily accompanied by a corresponding improvement in forelimb function [38]. Therefore, longitudinal monitoring using tests sensitive to the affected functional domain is essential for interpreting the nature, rather than merely the magnitude, of recovery [86,87].

Despite this extensive characterization of motor and sensorimotor recovery, cognitive and other non-motor outcomes were comparatively underrepresented across the included studies. Memory was assessed in only 29.4% of the studies [32,33,38,39,42,46,57,59,61,64], with spatial memory representing the most frequently investigated cognitive component, whereas recognition memory was evaluated in only one study [46]. Other non-motor domains were even less frequently explored, with anxiety and affective behavior assessed in only one study [32]. This imbalance represents an important gap in the preclinical assessment of post-stroke recovery, as functional recovery extends beyond sensorimotor performance and cognitive impairment represents an important component of post-stroke functioning and quality of life [97,98]. Notably, the limited representation of these outcomes cannot be attributed solely to short follow-up periods, since 50.0% of the included studies conducted behavioral assessments beyond two weeks [32,33,36,38,39,46,48,52,53,54,55,56,57,60,61,63,64]. Therefore, future studies using the photothrombotic model should incorporate broader multidomain behavioral assessments, including cognitive and non-motor outcomes, to provide a more comprehensive characterization of post-stroke recovery [98].

Histological characterization, in turn, showed a profile different from that observed in neuroprotection. Although structural assessment of the lesion was performed in 50.0% of the studies [33,38,41,45,47,48,52,63] and neuronal survival in 31.3% [33,41,45,52,53], processes associated with the reorganization of surviving tissue were also investigated, including angiogenesis and peri-infarct responses [52,53], neurogenesis/proliferation [33,52], neurotrophic factors [61], and neuronal or synaptic organization [41,47,53]. Thus, in recovery studies, histology is not restricted to the question of how much tissue was preserved but also contributes to determining which reorganization processes accompany functional evolution. Experimental evidence of dendritic, vascular, and corticospinal projection remodeling after stroke provides biological support for this interpretation [73,95,96].

However, five of the 16 recovery studies did not include histological characterization [39,54,55,56,64], limiting the association between the behavioral trajectory and its potential cellular or structural substrates. This issue is particularly relevant because behavioral recovery and neural tissue recovery do not necessarily follow parallel trajectories, and functional changes may result from reorganization of surviving circuits or compensation, even in the absence of proportional structural restitution [93,99]. Longitudinal integration of behavioral assessments with histology, neuroimaging, or physiological measures may therefore strengthen the distinction among restitution, neural reorganization, and compensation.

Taken together, the results indicate that the main contribution of the photothrombotic model to recovery studies lies in the possibility of relating a reproducible focal lesion to the longitudinal evolution of specific functions. In this context, evidence of recovery becomes more consistent when behavioral improvement is interpreted in relation to the functional domain assessed, the timing of the intervention, and the processes of plasticity or reorganization of surviving tissue, avoiding the automatic interpretation of improved performance as neural restitution.

### 4.5. Experimental Outcomes and Strength of Therapeutic Evidence

The results of this review showed that the correspondence between functional outcomes and mechanistic evidence varied across neuroprotection, neuromodulation, and post-stroke recovery, reflecting differences in the underlying purpose of the interventions. In neuroprotection, the high frequency of structural lesion characterization (66.7%) [32,40,42,51,58,62], together with assessments of neuronal survival [40,51,58], oxidative stress [58], inflammation [49], and cell death [62], allowed functional effects to be related to preservation of the neural substrate. This convergence is particularly relevant because reductions in infarct volume and behavioral improvement do not necessarily follow proportional trajectories, meaning that isolated histological changes may overestimate the functional relevance of an intervention [88,93].

In neuromodulation, the strength of the evidence depended less on the isolated demonstration of tissue preservation and more on the ability to relate the intervention to cellular, physiological, or circuit-level changes. Glial responses (60%) [35,37,44,46,59,60], cell death/apoptosis (50%) [37,44,46,59,60], and neuronal survival (40%) [36,44,46,60] were complemented, in some studies, by neuroimaging, perfusion measurements, or electrophysiological assessments [36,37,44,50,57,59,60,65]. This integration provides mechanistic support for the behavioral response and is particularly relevant for demonstrating target engagement, since functional improvement following stimulation does not, by itself, establish that the intended neural circuit or process was effectively modified [84,91].

In recovery studies, interpretation of the outcomes presented a distinct issue. The predominance of longer follow-up periods favored characterization of the functional trajectory; however, five of the 16 studies did not include histological assessment [39,54,56,64]. Although other studies investigated neurogenesis, angiogenesis, neurotrophic factors, and neuronal or synaptic organization [33,41,47,52,53,61], the association between these changes and functional performance was not uniformly established. This dissociation is relevant because behavioral improvement over time may result from both restitution and circuit reorganization or from compensatory strategies, processes that do not necessarily have the same biological or therapeutic significance [24,99].

Complementary assessments contributed to reducing these interpretative gaps. Biochemical and molecular analyses were employed in different studies [37,39,40,42,44,45,46,49,51,52,59,60,62,64], whereas MRI [34,43,49,50,54,55,56], PET/CT [34,36,50], perfusion measurements [37,43,44,60], and electrophysiological assessments [57,65] provided additional information on brain structure, hemodynamics, and neural function. However, the value of these approaches lies not in the number of techniques added to the protocol, but in their ability to test a biologically relevant step between the intervention and the functional outcome. Thus, molecular, histological, or physiological changes become more informative when their relationship with the therapeutic hypothesis can be established.

### 4.6. Limitations and Future Perspectives

This systematic review presents some limitations that should be considered when interpreting the findings. First, limitations inherent to the photothrombotic stroke model should be considered when assessing its translational relevance. Although photothrombosis provides precise spatial control and high reproducibility of ischemic lesions, stroke induction results from photoactivation of a photosensitive dye, generating reactive oxygen species that promote endothelial injury, platelet aggregation, and local microvascular thrombosis [17,27]. This mechanism differs from the pathophysiology of most human ischemic strokes, which commonly involve thromboembolic occlusion of cerebral arteries. In addition, conventional photothrombotic lesions are characterized by a limited ischemic penumbra and collateral blood flow, restricting the investigation of potentially salvageable tissue and reperfusion-related processes that are central to acute ischemic stroke [17,81]. This limitation is particularly relevant to neuroprotection studies, as many neuroprotective strategies are intended to preserve potentially salvageable tissue within the ischemic penumbra. Furthermore, because conventional photothrombosis produces persistent microvascular occlusion rather than controlled transient occlusion followed by reperfusion, it is not well suited for investigating reperfusion injury or therapies specifically targeting reperfusion-associated mechanisms [17,81]. Therefore, although the model is particularly useful when precise lesion localization and reproducibility are required, these characteristics should be considered when extrapolating findings to human thromboembolic stroke and when selecting the model according to the specific biological or therapeutic question.

Incomplete reporting of anesthetic protocols, particularly post-anesthetic recovery time, further limited the assessment of anesthesia as a potential source of variability in therapeutic and behavioral outcomes.

Because eligibility required a therapeutic intervention and behavioral assessment, purely methodological studies investigating photothrombotic induction parameters or model reproducibility were not included. Therefore, the induction parameters summarized here should be interpreted as representative of therapeutic and functional studies rather than of the entire photothrombotic stroke literature.

The analysis was restricted to the photothrombotic model of ischemic stroke in rats, which allowed a more focused comparison of experimental protocols but limits the generalization of the results to other species and stroke induction models. Furthermore, as discussed above, the predominance of young adult male Sprague-Dawley rats and the limited representation of females, aged animals, and clinically relevant comorbidities further restrict the external validity of the available evidence. In addition, heterogeneity in photothrombosis parameters, therapeutic strategies, follow-up periods, behavioral tests, and complementary assessments limited direct comparisons among studies. The predominance of motor and sensorimotor assessments, together with the limited evaluation of cognitive and other non-motor outcomes, also restricts a comprehensive assessment of post-stroke recovery. Incomplete reporting of some methodological parameters also restricted a more detailed assessment of protocol reproducibility. Future studies should therefore combine improved reporting and standardization of photothrombotic induction parameters with greater biological diversity and broader multidomain functional assessments. In addition, the selection of the photothrombotic model should be guided by the specific biological and therapeutic question under investigation, particularly when processes involving penumbral tissue, reperfusion, or thromboembolic mechanisms are central to the study.

## 5. Conclusions

This systematic review showed that the utility of the photothrombotic model of ischemic stroke in rats depends on the therapeutic purpose being investigated. Although there is relative convergence in general aspects of model induction, variability in irradiation parameters and their incomplete reporting remain important issues for study reproducibility.

Analysis according to therapeutic purpose revealed three distinct experimental profiles. In neuroprotection, the model was mainly used within early time windows, in which the association between molecular mechanisms, neuronal preservation, and behavioral performance allows assessment of whether limitation of injury translates into functional benefit. In neuromodulation, the ability to produce spatially controlled lesions favored the investigation of interventions targeting remaining neural circuits, with particular relevance given to the integration of behavioral outcomes with measures of neural activity, perfusion, neuroimaging, or cellular responses. In post-stroke recovery, longer follow-up periods and greater use of tests assessing dexterity, asymmetry, and motor performance allowed investigation of functional evolution, while histological and complementary assessments contributed to relating these changes to plasticity and reorganization of surviving tissue.

Thus, the main contribution of this review is to demonstrate that neuroprotection, neuromodulation, and recovery should not be investigated using the same experimental rationale. The selection of the temporal window, behavioral tests, and histological or complementary assessments should reflect the biological process intended to be modified. This relationship among therapeutic purpose, experimental design, and outcomes provides a framework for selecting and interpreting photothrombotic protocols more consistently in future preclinical stroke studies.

## Figures and Tables

**Table 1:** Aspects of the induction of the photothrombosis stroke model.

		Animal				Photosensitizer Characteristics (Rose Bengal)		Ischemic Model Preparation			Irradiation Parameters			
Study	Year	Linage	Sex	Weight (g)	Age (Weeks)	Dose (mg/kg)/Conc. (mg/mL)	Adm. Route	Craniotomy	Injury Site	Delay Photosensitizer-Laser	Irradiation Source	Power (Device/Applied)	Application Time (Min)	Distance to Target (mm)
Li et al. [32]	2025	Sprague-Dawley	Male	300 ± 20	8	100/100 g/0.1 mL	IP	No	B-Ctx	40 min before surgery	LED (565 nm); Laser (473 or 593 nm)	NR/NR	20	1 mm above the rat’s skull
Beuker et al. [33]	2025	Wistar	Male	NR	12–13	1.33/10	IV—Femoral vein	No	Right FrCx	2 min	Laser (561 nm; Ø 8 mm)	NR/NR	20	NR
Luo et al. [34]	2024	Sprague-Dawley	Male	300–350	8–12	42.9–50/15	IV—Tail vein	Yes	U-M1/S1	2 min	Laser (532 nm; Ø 3.5 mm)	NR/25 mW	15	NR
Fritsch et al. [35]	2024	Sprague-Dawley	Male	309–400	8.5 ± 0.5	9.975/NR	IV—Tail vein	No	Left M1	2 min	Cold light	150 W/NR	20	NR
Kim et al. [36]	2024	Sprague-Dawley	Male	280–300	8	20/NR	IV—Tail vein	Yes	Ctr PLIC	NR	Laser (532 nm; OF core Ø 62.5 µm)	NR/3.7 mW	1.5	7.8 mm deep into the PLIC
Feng et al. [37]	2024	Sprague-Dawley	Male	NR	8	100/NR	IP	No	B-Ctx	5 min	Cold light (Ø 6 mm)	NR/NR	15	NR
Caine et al. [38]	2024	Sprague-Dawley	Male	NR	16	10/NR	IV—Tail vein	Skull thinning	Ctr MCx	0 min	Laser (532 nm)	NR/46.4 mW	10	NR
Song et al. [39]	2024	Sprague-Dawley	Male	NR	10	50/NR	IV—Femoral vein	No	Left SMC	0 min	Light source	150 W/NR	10	NR
Lan et al. [40]	2024	Sprague-Dawley	Male	200 (mean)	8	50/NR	IV—Tail vein	Yes	U-M1	NR	Cold light	NR/NR	25	NR
Hakon et al. [41]	2024	Sprague-Dawley	Male	NR	NR	0.5 mL/10	IV	No	Left SMC	10 min	Cold light (GF 515 nm)	NR/NR	20	NR
Choi et al. [42]	2024	Wistar	Male	280.45 ± 2.25	8	10/NR	IP	No	Right M1	5 min	Cold light (Ø 4 mm)	NR/NR	20	NR
Babenko et al. [43]	2024	NR	NR	NR	NR	40/30	IV—Jugular vein	No	B-Ctx	NR	Laser (520 nm; Ø 3 mm)	NR/100 mW	15	NR
Suo et al. [44]	2023	Sprague-Dawley	Male	250–300	8–10	50/NR	IV—Tail vein	Yes	Left SMC	NR	Laser (532 nm)	NR/80 mW	15	NR
Shin et al. [45]	2022	Sprague-Dawley	NR	NR	Neonate	25/NR	IP	No	Right Ctx	30 min before surgery	LED (560 nm)	NR/NR	1	NR
Zong et al. [46]	2022	Sprague-Dawley	Male	NR	12	100/NR	IP	No	B-Ctx	5 min	Cold light (Ø 6 mm)	150 W/NR	15	In contact with the skull
Vitrac et al. [47]	2022	Sprague-Dawley	Male	NR	9–10	10/40	IV—Tail vein	Yes	Ctr M1	0 min	Cold light	NR/NR	20	NR
Nucci et al. [48]	2022	Wistar	Male	250–300	8	NR/50	IP	Yes	Left S1	20 min	Light source (Ø 50 mm)	NR/NR	65	5 mm from dura mater
Li et al. [49]	2022	Sprague-Dawley	Male	NR	24	15/NR	IP	No	NR	10 min	Cold light (Ø 4 mm)	NR/NR	15	NR
Krämer et al. [50]	2022	Wistar	Male	321 ± 14	8–10	14.9–16.3 mL/10	IV	No	Ctr M1/CFA	NR	Cold light (Ø 9 mm)	NR/NR	15	NR
Deng et al. [51]	2022	Sprague-Dawley	Male	200–230	8	50/20	IV—Tail vein	Yes	U-M1	NR	Laser (560 nm; Ø 1.5 mm)	NR/NR	25	NR
Yew et al. [52]	2021	Sprague-Dawley	Male	270–340	NR	13/10	IV—Femoral vein	No	Ctr CFA	0 min	Light source (GF 532.5–587.5 nm; Ø 6 mm)	150 W/NR	15	Close as possible to the skull without contacting it
Yew et al. [53]	2021	Sprague-Dawley	Male	295–331	NR	13/10	IV—Femoral vein	No	Ctr CFA	0 min	Light source (GF 532.5–587.5 nm; Ø 6 mm)	150 W/NR	15	Close as possible to the skull without contacting it
van Lieshout et al. [54]	2021	Sprague-Dawley	Male	326 ± 26	10–11	NR/25	IV—Saphena	No	Ctr SMC	NR	Cold light	NR/NR	20	NR
Sinke et al. [55]	2021	Sprague-Dawley	Male	423 ± 16 425 ± 19 e 426 ± 21	11	25/NR	IV—Saphena	No	Right SMC	0 min	Cold light (Ø 4.5 mm; GF 515 nm)	NR/NR	20	NR
Li et al. [56]	2021	Sprague-Dawley	Male	230–280	8–12	53.6–65.2/15	IV—Tail vein	Yes	Ctr Ctx	2 min	Laser (532 nm)	NR/25 mW	15	NR
Gao et al. [57]	2021	Sprague-Dawley	Male	300–350	Adult	42.9–50/15	IV—Tail vein	Yes	Right CFA	2 min	Laser (532 nm; Ø 3.5 mm)	NR/25 mW	15	NR
Yi et al. [58]	2020	Sprague-Dawley	Both	NR	Neonate	25/5	IP	Yes	U-SMC	At least 30 min before	Laser (532 nm)	36 mW/NR	1	NR
Zong et al. [59]	2020	Sprague-Dawley	Male	200–250	NR	100/NR	IP	No	U-Ctx	5 min	Cold light (Ø 6 mm)	NR/NR	15	NR
Zong et al. [60]	2020	Sprague-Dawley	Male	NR	12	100/NR	IP	No	U-Ctx	5 min	Cold light (Ø 6 mm)	NR/NR	15	NR
Song et al. [61]	2020	Sprague-Dawley	Male	300–350	10	50/NR	IV—Left femoral vein	No	Left SMC	NR	Light source	150 W/NR	10	NR
Rahman et al. [62]	2020	Sprague-Dawley	Male	250–350	8–12	20/NR	IV—Tail vein	No	Right SMC	NR	Cold light	150 W/NR	20	NR
Matwee et al. [63]	2020	Sprague-Dawley	Male	NR	12	10/NR	IV—Tail vein	Skull thinning	Ctr CFA	NR	Laser (532 nm)	NR/25.2–27 mW	10	NR
Joa et al. [64]	2020	Sprague-Dawley	Male	250–300	8	10/NR	IP	No	Ctr SMC/M1	NR	Light source (Ø 6 mm)	NR/NR	20	Close as possible to the skull without contacting it
Boddington et al. [65]	2020	Wistar	Male	NR	6–9	1.3/NR	IV—Jugular vein	Yes	Left CFA	2 min	Cold light	4–5 W/NR	20	NR

Abbreviations. NR: not reported; Conc.: concentration; Adm.: administration; IV: intravenous; IP: intraperitoneal; MCx: motor cortex; PLIC: posterior limb of the internal capsule; S1: primary somatosensory cortex; FrCx: frontal cortex; M1: primary motor cortex; SMC: sensorimotor cortex; Ctx: cerebral cortex; Ctr: Contralateral; U-: unilateral; B-: bilateral; CFA: caudal forelimb area; HPC: hippocampus; Right: right hemisphere; Left: left hemisphere; Ø: beam diameter; LED: light-emitting diode; OF: optical fiber; GF: green filter; g: gram; mg: milligram; kg: kilogram; mL: milliliter; min: minute; mm: millimeter; nm: nanometer; W: watts; mW: milliwatt.

**Table 2:** Aspects of the Evaluation of the Photothrombotic Stroke Model.

Study	Study Purpose	Experimental Groups (*n*)	Follow-Up	Behavioral Assessment		Histological Analysis	Extra Assessments	Outcomes
				Behavioral Test	Evaluation Frequency			
Neuromolulation								
Fritsch et al. [35]	Neuromolulation for acute stroke recovery	Sh tDCS (6); anodal tDCS 0.8 A/m^2^ (5), 31.8 A/m^2^ (8) or 47.8 A/m^2^ (7)	48 h AS	mNSS; pasta handling task	mNSS: 1 d BS, 1 and 2 d AS; pasta handling: 1 d BS and 2 d AS	FJ-C; IHQ: Iba1	NR	Low-intensity anodal tDCS applied to the ipsilesional forelimb motor cortex 24 h after photothrombotic stroke in awake rats was safe.
Kim et al. [36]	Neuromolulation for chronic stroke recovery	Con (8); CamKII- Sh (7); PV-Sh (8); hSyn-Sh (7); CamKII-ChR2 (8); PV-ChR2 (8); hSyn-ChR2 (8)	28 d AS (35 d after virus)	SPRT	Every day until euthanasia	IF: GFP, CamKII, PV, NeuN, BDNF	Micro-PET/CT	Optogenetic CS modulation of PV-expressing neurons promoted post-stroke recovery. The PV-ChR2 group showed activation of the corticostriatal circuit, which was associated with functional recovery following capsular infarcts, increased plasticity (indicated by BDNF expression), and reduced diaschisis, as reflected by behavioral outcomes.
Feng et al. [37]	Neuromolulation for stroke recovery	Sh (5–6); FL (5–6); PT (5–6); PT + PBM (5–6); PT + PBM + FL (5–6)	7 d AS	Cylinder test; ART; WHT; BWT; LRWT	1 d BS; 1 d and 7 d AS	EBD; IF: testosterone, CD31, RECA-1, 3β-HSD1, 17β-HSD5, ZO-1, Claudin 5, cleaved caspase-3, GFAP, Iba1, MAP2, FITC- dextran; TUNEL	LSCI; western blot; ELISA	PBMT attenuates cerebrovascular injury and behavioral deficits associated with testosterone/AR following ischemic stroke.
Suo et al. [44]	Neuromolulation for acute stroke recovery	Sh/NS (6); Sh/S (6); PT/NS (15); PT/S (15); PT/S/Sh (15); PT/S/Sh (15)	3 d AS	mNSS; LRWT	1 d and 3 d AS	Cresyl violet; IF: ZO-1, Occludin, CD31, IL-10, IL-10R, NeuN, MAP2, GFAP, CD11b, MMP2, MMP9, caspase-3, cleaved caspase-3; IHC: IgG; TUNEL	Western blot; RT-qPCR; gelatin zymography assay	Optogenetic photostimulation of astrocytes preserved BBB integrity, attenuated neuronal apoptosis, and improved neurobehavioral outcomes in acute stroke through IL-10/MMP2 signalling
Zong et al. [46]	Neuromolulation and neuroprotection for stroke recovery	Con; PT; PT + cTBS	6, 22 and 35 d AS	FPT; BWT; NOR;	22 d AS	IHC: DCX, p-TrkB; Nestin, SOX2, Tuj1, NeuN, Olig2, FGF2, BDNF, MBP, BrdU, Iba1, GFAP; TUNEL	Western Blot	cTBS therapy promoted functional recovery after stroke, enhanced early and delayed neurogenesis, increased the NPCs, DCX-positive neurons in the cortical peri-infarct, reduced reactive gliogenesis, and promoted oligodendrogenesis and myelin preservation.
Krämer et al. [50]	Neuromolulation for stroke recovery	Sh-stimulated (10); stimulated (10); PET/CT group (6)	3 (PET) and 7 d (MRI) AS	SPRT	30 d-trainning; 1 d BS; 2 and 7 d AS	H&E	PET/CT; MRI	Ipsilateral STN-HFS improved forelimb dexterity in rats when 7 daily applied and increased glucose metabolism in the corticosubthalamic and pallidosubthalamic pathways, reduced infarct size, spared periinfarct neurons, and promoted motor recovery after stroke.
Gao et al. [57]	Neuromolulation for stroke recovery	Electrodes (5); S + electrodes (4)	NR	Lever press task	3 d-trainning 4 months AS, tests during 3 w AS	NR	Neural Signals and Force Data Collection	High-frequency LFPs power accurately decode force signals and showed long-term stability, supporting its potential for BMI-based motor function reconstruction after chronic stroke
Zong et al. [59]	Neuromolulation for stroke recovery	Con (8–10); S (8–10); S + rTMS (8–10)	5 and 21 d AS	ART; LRWT; WHT	1, 3, 5, 7 and 13 d AS	Cresyl violet; FJ-C; DHE staining; IF: Synaptophysin, spinophilin, 4-HNE, 8-OHdG, MAP2, GFAP, C3d, S100A10, Iba1, cleaved-IL-1β, cleaved caspase-3, phospho-Histone H2A.X, TGF-β, MDA, TUNEL;	LDF; Western Blot; NADPH Oxidase Activity and Superoxide Production; Cytokine array; Caspase activity	Theta-burst rTMS provided robust neuroprotection when applied at least 3 h after ischemic stroke, attenuated behavioral deficits and infarct volume, and reduced synaptic loss and neuronal degeneration in the peri-infarct cortex, particularly at later stages of stroke.
Zong et al. [60]	Neuromolulation for stroke recovery	Con (NR); S (NR); S + rTMS (NR)	6 and 22 d AS	Grip strength test; PHR	Grip strength: baseline BS and 22 d AS; PHR: 22 d AS	EBD; IF: IgG, pimonidazole, VEGF, CD31, Ki67, MBP, NeuN, RECA-1, BrdU, PDGFRβ, TGF-β, GFAP, C3d, S100A10, synaptophysin, spinophilin, HIF-1α, FITC-dextran; IHC: IgG, RECA-1; TUNEL; PLA	LDF; Western Blot; NADPH Oxidase Activity and Superoxide Production; Cytokine array;	rTMS promoted peri-infarct microvascular repair after stroke, enhanced HIF-1α signaling and A2 astrocytic polarization, promoted long-term angiogenesis, reduced endothelial cell apoptosis, and preserved neuronal and synaptic integrity and behavioral outcomes
Boddington et al. [65]	Neuromolulation for stroke recovery	Sh; cTBS; iTBS (early/delayed; minimal/intensive)	After finishing all electrophysiological experiments	Grid-walking task	NR	Cresyl violet	In vivo electrophysiology	High- and low-dose intermittent theta-burst rTMS improved recovery to a similar extent when applied 10 days after stroke, with recovery positively correlated with ipsilesional excitability. Contralesional continous rTMS did not alter rocovery but reduced ipsilesional excitability.
Neuroprotective effect								
Li et al. [32]	Neuroprotective effect in acute stroke recovery	Sh (9); Veh (7); SAA (7)	24 h (TTC); 8 d (Nissl); 60 d (behavioral tests)	MWM; BWT; treadmill test; OFT; EPM; SPT	MWM: 4 d-trainning, 20 d-AS reversal learning and 60 d-AS double reversal learning; Other tests: NR	TTC; Nissl	PET	SAA (2 mg/Kg) remained effective in reducing infarct volume when administered up to 8 h after stroke, with efficacy dependent on treatment timing
Lan et al. [40]	Neuroprotective effect in stroke recovery	Exp. 1: Con (6); Sh (6); ischemia (6); Mus (6); Exp. 2: Con (6); Sh (6); ischemia (6); Mus (6); L-NAME (6)	24 h AS	OFT	1 d AS	Nissl; IHC: NeuN;	ELISA; western blot; measurement of oxidative stress indexes; TEM; measurement of NO and NOS contents	GABAAR activation exerts a neuroprotective effect during cerebral ischemia, by enhancing antioxidant capacity, reducing oxidative products, suppressing autophagy via AMPK-mTOR pathway, and inhibiting apoptosis via Bax/Bcl-2 pathway, and implicating GABAAR-mediated autophagy signaling.
		Exp. 3: Sh (6); ischemia (6); rapamycin (6); 3-MA (6)						
Choi et al. [42]	Neuroprotective effect in stroke recovery	Sh (8); S (8); S + TST (8); S + 5-Aza-dC (8); S + 5-Aza-dC + TST (8)	13 d AS	Staircase test; cylinder test	Staircase: 7 d-trainning BS	Cresyl violet	RNA Isolation and RNA-Sequencing; qPCR	Post-stroke rNPFF treatment regulated cellular stress responses, synaptic plasticity, and energy homeostasis, thereby promoting neuronal survival and recovery.
					4, 5 e 12 d após AVC			
Babenko et al. [43]	Neuroprotective effect in stroke recovery	Con (10); PT (15); PT + tPA (13); PT + tPA + LiCl (8)	7 d AS	FPT; cylinder test	FPT: 3 and 6 d AS; cylinder: 7 d AS	EBD	MRI	Post-stroke Li+ treatment improved neurological outcomes and increased BBB integrity after thrombolytic therapy
Zong et al. [46]	Neuromolulation and neuroprotection for stroke recovery	Con; PT; PT + cTBS	6, 22 and 35 d AS	FPT; BWT; NOR;	22 d AS	IHC: DCX, p-TrkB; Nestin, SOX2, Tuj1, NeuN, Olig2, FGF2, BDNF, MBP, BrdU, Iba1, GFAP; TUNEL	Western Blot	cTBS therapy promoted functional recovery after stroke and enhanced and sustained neurogenesis at early and delayed atages, expanding the NPCs, DCX-positive immature and mature neurons in the peri-infarct cortex, reducing reactive gliogenesis, promoting oligodendrogenesis and preserving myelination.
Li et al. [49]	Neuroprotective effect in perinatal stroke recovery	MND sham (8); MND S (8); MND S + FGF21 (8); MHFD Sh (8); MHFD S (8); MHFD S + FGF21 (8)	After behavioral tests were completed	Grip strength test; BWT	1, 2 and 3 d AS	IF: NF-κB, GFAP	Western blot; MRI; RT-qPCR; blood index (ELISA and biochemical analyzer); transcriptome sequencing	MHFD altered astrocytes characteristics and transcriptomic profiles in offspring, and was associated with poorer stroke outcomes. FGF21 exerted neuroprotective effects, by inhibiting NF-κB, inflammatory factors, and activating the PI3K/Akt pathway, with more astrocytes observed in controls than in the MHFD group.
Deng et al. [51]	Neuroprotective effect in stroke recovery	Con (6); Sh (6); S (6); L-NAME (6); L-NAME + S (6)	2, 6, 12 and 24 h AS	OFT	1 d AS	Nissl; IHC: NeuN	Oxidation and Antioxidant Test; measurement of NO and NOS content	L-NAME and Leo treatment protected against oxidative stress and neuronal apoptosis in cerebral ischemia by inhibiting the NO/NOS system. Leo treatment decreased ROS and MDA levels and increased SOD activity and GSH content in PC12 cells exposed to OGD.
Yi et al. [58]	Neuroprotective effect in stroke recovery	Exp.1: Con (6); ASA (6); Clop (5); CoQ10 (5); Exp.2: Con (5); ASA (5); Clop (5); CoQ10 (5)	24 h and 7 d AS	WHT	1 d BS, immediately AS and 2 d and 5 d AS	TTC; DHE Staining; IHC: Iba1, GFAP, NeuN	NR	ASA, Clop, and CoQ10 treatment before and after stroke reduced stroke lesions and increased behavioral activity, suggesting neuroprotective effects in neonatal stroke.
Rahman et al. [62]	Neuroprotective effect in stroke recovery	Con (8); Sh (8); normal-sheep IgG (8); S + anti- proBDNF IgG (8)	7 d AS	Cylinder test; rotarod; ART; corner test	1 d BS and 7 d AS	H&E; cresyl violet; IHC: proBDNF, GFAP, Iba1; TUNEL	Western blot	Anti-proBDNF treatment reduced infarct size and apoptotic signaling proteins, promoted cellular growth, and improved sensorimotor function 7 days after ischemic insult.
Post-stroke Recovery								
Beuker et al. [33]	Recovery and neurogenesis in subacute and chronic stroke	Sh (7); Veh (14); acylated ghrelin (14); ghrelin receptor antagonist (14)	28 d AS	Neuroscore; rotarod; ART; cylinder test; MWM	Baseline: 1 d BS; Rotarod: 5 d-trainning BS; ART: 3 d-trainning BS; MWM: 4 d-trainning AS;	IHC: NeuN, CD31, CldU, IdU, nestin; Toluidine blue	NR	Ghrelin promoted acute neurogenesis in the dentate gyrus and peri-infarct area, enhancing long-term neuronal survival and functional recovery.
					All tests were performed during 28 d AS			
Luo et al. [34]	A PET radiopharmacy for neurological recovery assessment	Saline (8); safinamide (11)	NR	Cylinder test; FPT	Until 14 d AS	Golgi-Cox staining; IHC: GFAP, GLUT1, Iba1	MRI; Micro-PET/CT	[18F]SynVesT-1 PET effectively quantified the spatiotemporal dynamics of synaptic density in the rat model of stroke, as a potential non-invasive biomarker.
Caine et al. [38]	ER for stroke recovery	Standard care (16); ER (16)	35 d AS	Staircase test; cylinder test	Montoya: 4 d-trainning BS and 5–6, 21–22 and 33–34 d AS; cylinder test: 6, 22 and 34 d AS	Cresyl violet; IF (muscle): BA-F8, SC-71, BF-F3, 6HI	NR	While the modified ER did not promote forelimb motor recovery, it may be useful for studying the role of skeletal muscle plasticity in post-stroke recovery.
Song et al. [39]	Intensity exercise for stroke recovery and neurogenesis marker	No exercise (9); treadmill 18 m/min (9); treadmill 24 m/min (9); treadmill 30 m/min (9)	30 d AS	MWM	3 d-trainning 1 week BS; 1 test 1 d AS and 30 d AS	NR	RNA Sequencing; western blot	Moderate-intensity exercise may contribute to neuroplasticity. NF-κB is the key neurotrophin for neurogenesis related to exercise intensity.
Hakon et al. [41]	Post acute stroke recovery	Sh Veh (9); Sh MTEP (8); S + Veh (9); S + MTEP (8)	14 d AS	PPT; BWT; grip strength test; PHR; ART;	BWT: 3 d-trainning BS; all tests were perfomed on stroke day, 2, 7 and 14 d AS	IHC/IF: NeuN, PV, chondroitin sulphate proteoglycan, GAD67	NR	Inhibition of mGluR5 with NAMs in the post-acute phase after stroke accelerated functional brain reorganization and recovery of sensorimotor functions without reducing brain damage.
Shin et al. [45]	Perinatal acute stroke recovery	Con + NC NP (6); Con + PER NP (6); RB + NC NP (6); RB + PER NP (6)	24 h AS	WHT	1 d BS and 1 d AS	TTC; H&E; cresyl violet; IHC: Iba1, NeuN, GFAP; TUNEL	qPCR; western blot	PER-loaded PLGA nanoparticles (intrathecal targeting of microglia) decreased infarct size and improved motor function in rat model of perinatal ischemic stroke.
Vitrac et al. [47]	Stroke recovery	Saline solution (18); dopamine antagonists (16)	12 d AS	SPRT; ART	SPRT: pre-trainning + 3 d until 12 d AS; ART: 1, 5 and 8 d AS	Nissl; IHC: TH	NR	DA transmission in the peri-infarct cortex is crucial for motor skill recovery after stroke, suggesting a common mechanism between learning and recovery
Nucci et al. [48]	Stroke recovery	Sh (5); S (5); S + MSC (5); S + exercise (5); S + MSC + exercise (5)	24 h AS (TTC), 28 d AS	Cylinder test; OFT	2 d BS; 7, 14, 21 and 28 d AS	TTC	LSCI (PeriCam)	Combined MSC therapy and physical activity resulted in greater post-stroke recovery than either therapy alone, with the most pronounced benefits observed in the acute phase of stroke.
Yew et al. [52]	Stroke recovery	Veh (24); olomoucine (24)	3, 7 and 29 d AS	FPT; SPRT	FPT: 3 d-habituation BS, tests on 0.12, 1, 7, 14, 21 and 28 d AS; SPRT: 15 d-trainning BS, baseline 4 d AS, tests on 1, 7, 14, 21 and 28 d AS	Cresyl violet; IHC: Iba1, CD68, Ki67, NeuN, GFAP, collagen IV	Western blot	Olomoucine treatment did not affect infarct volume or affected forelimb in the placing test, but improved recovery in a skilled reaching test, produced small changes in Iba1 immunolabelling and in the number of CD68-positive cells in cerebral cortex, and did not selectively modify responses in peri-infarct tissue.
Yew et al. [53]	Stroke recovery	Con (9); DPSC (9)	28 d AS	FPT; SPRT; cylinder test	FPT: 0.12, 1, 7, 14, 21, 28 d AS; SPRT: 1–28 d AS; cylinder: 1, 7, 14, 21, 28 d AS	IHC: GFAP, collagen IV, synaptophysin, human mitochondrial protein, NeuN	NR	DPSC treatment promoted recovery without modifying initial infarct formation; the decrease in reactive astrogliosis in peri-infarct tissue contributed to these changes by promoting adaptive responses in neighboring neurons.
van Lieshout et al. [54]	Translational behavior stroke recovery	Sh-stimulation (NR); rTMS (NR)	23 d AS	SPRT	1 d BS and 3, 9, 16, 23 d AS	NR	MRI	Post-stroke changes in skilled reaching performance are highly similar between rats and humans, offering an effective and translational means for assessing motor recovery in experimental and clinical stroke settings.
Sinke et al. [55]	Stroke degeneration	Con 1 w (7); S 1 w (8); Con 8 w (8); S 8 w (7); Con 28 w (8); S 28 w (7)	NR	Cylinder test	1 d BS and 1 d, 1, 3, 5, 8, 12, 18 and 28 w AS	NR	Resting-state fMRI (rs-fMRI); diffusion tensor imaging and tractography	Post-stroke motor recovery (at week 28) is possible despite degeneration of the CST (up to week 8) and may be supported by intact transcallosal tract integrity, allowing neuronal communication between hemispheres.
Li et al. [56]	Stroke degeneration	NA	30 d AS	SPRT	3 d-trainning BS, tests on 1 d BS; 2–4, 6, 8, 10, 12, 14, 16, 18, 20, and 21 d AS	NR	Ex vivo MRI	Focal ischemic lesions may induce remote WM degeneration, but this is limited to fibers connected to the primary lesion through perilesional, interhemispheric, and subcortical axonal connections. So, primary lesion volume might be the determining factor for motor function deficits.
Song et al. [61]	Stroke recovery	Non-exercise (9); early submaximal (9); early maxima (9); late submaximal (9); late maximal (9)	NR	MWM	3 d-trainning BS, tests on 1 d and 30 d AS	IHC: BDNF	Biochemical analysis	Early submaximal swimming exercise was the most effective treatment for recovering neurological deficits in a rat model of cerebral infarction
Matwee et al. [63]	Stroke recovery	Con- Sh (6); PEM- Sh (6); Con-ISCH (8); PEM-ISCH (6)	30 d AS	Cylinder test	Baseline 2 d BS, tests on 4, 16 and 29 d AS	Cresyl violet	Biochemical analysis	Preventing post-stroke PEM (low protein) enhanced motor recovery during the chronic phase.
Joa et al. [64]	Stroke recovery	RG (12); SG (12)	28 d AS	SPRT; RR; RMT	SPRT: baseline diarly per 3 w BS, tests on 7, 14, 21 and 28 d AS; RR: 3 d-trainning BS, tests on 1, 7, 14, 21 and 28 d AS;	NR	Western blot	Aerobic exercise inhibited tau phosphorylation and acetylation and modulated the expression of tau-related proteins after stroke by modifying the p70-S6K and p-AMPK pathways.
					RMT: 3 d-habituation BS, tests on 1, 7, 21 and 28 d AS			

Abbreviations. PET: positron emission tomography; ER: enriched rehabilitation; Sh: sham; tDCS: transcranial direct current stimulation; Con: control; CamKII: calcium/calmodulin-stimulated protein kinase II; hSyn: human synapsin 1 gene; ChR2: channelrhodopsin-2; PV: parvalbumin; FL: flutamide; PT: photothrombotic; PBM: photobiomodulation; NS: non stroke; S: stroke; cTBS: continuous theta-burst stimulation; CT: computed tomography; rTMS: repetitive transcranial magnetic brain stimulation; NR: not reported; iTBS: intermittent theta-burst stimulation; Veh: vehicle; SAA: salvianolic acid A; Exp.: example; Mus: muscimol; L-NAME: L-nitroargininemethyl ester; Leo: leonurine; 3-MA: 3-Methyladenine; TST: task-specific training; 5-Aza-dC: 5-aza-2′-deoxycytidine; tPA: tissue plasminogen activator; LiCl: lithium chloride; MND: maternal normal diet; FGF21: fibroblast growth factor 21; MHFD: maternal high-fat diet; ASA: Aspirin; Clop: clopidogrel; CoQ10: Coenzyme Q10; IgG: Immunoglobulin G; proBDNF: pro brain-derived neurotrophic factor; MTEP: (3-[(2-methyl-1,3-thiazol-4-yl)ethynyl]pyridine hydrochloride); NC: negative control; NP: nanoaprticles; PER: perampanel; RB: rose bengal; MSC: mesenchymal stem cell; DPSC: dental pulp stem cells; NA: not applicable; PEM: protein-energy malnutrition; ISCH: induction of cortical stroke; RG: rehabilitation group; SG: sedentary group; AS: after stroke; MRI: Magnetic Resonance Imaging; TTC: 2,3,5-Triphenyl tetrazolium chloride; mNSS: modified neurological severity score; SPRT: single pellet reaching task; ART: adhesive removal test; WHT: Wire Hanging Test; BWT: beam walking test; LRWT: ladder rung walking test; FPT: forelimb placing test; NOR: novel object recognition; PHR: postural hang reflex; MWM: morris water maze; OFT: open field test; EPM: elevated plus maze; SPT: sucrose preference test; PPT: paw placement test; RR: rotarod; RMT: Radial maze test; BS: before stroke; FJ-C: fluoro-jade C; IHC: immunohistochemistry; Iba1: Ionized Calcium-Binding Adapter Molecule 1; IF: immunofluorescence; GFP: green fluorescent protein; NeuN: Neuronal Nuclei; BDNF: brain-derived neurotrophic factor; EBD: brain evans blue; CD: cluster of differentiation; RECA-1: rat endothelial cell antigen-1; 3β-HSD1: 3β-hydroxysteroid dehydrogenase type 1; 17β-HSD5: 17β-hydroxysteroid dehydrogenase type 5; ZO-1: zonula occludens-1; GFAP: glial fibrillary acidic protein; MAP2: microtubule-associated protein 2; FITC: fluorescein isothiocyanate; TUNEL: terminal deoxynucleotidyl transferase dUTP nick end labeling; IL10: interleukin-10; MMP2: matrix metalloproteinase-2; MMP9: matrix metalloproteinase-9; DCX: doublecortin; p-TrkB: phosphorylated tropomyosin receptor kinase B; SOX2: SRY-box transcription factor 2; Tuj1: class III β-tubulin; Olig2: oligodendrocyte transcription factor 2; FGF2: fibroblast growth factor 2; MBP: myelin basic protein; BrdU: 5-bromo-2′-deoxyuridine; H&E: Hematoxylin and Eosin; DHE: dihydroethidium; 4-HNE: 4-hydroxynonenal; 8-OHdG: 8-hydroxy-2′-deoxyguanosine; C3d: Complement component 3 fragment d; S100A10: S100 calcium-binding protein A10; cleaved-IL-1β: cleaved interleukin-1 beta; Phospho-histone H2A.X: Phosphorylated histone H2A.X; TGF-β: transforming growth factor beta; MDA: malondialdehyde; VEGF: vascular endothelial growth factor; PDGFRβ: platelet-derived growth factor receptor beta; HIF-1α: hypoxia-inducible factor 1-alpha; PLA: proximity ligation assay; NF-κB: nuclear factor kappa-light-chain-enhancer of activated b cells; PI3K: phosphoinositide 3-kinase; Akt: protein kinase B; CldU: 5-chloro-2′-deoxyuridine; IdU: 5-iodo-2′-deoxyuridine; GLUT1: glucose transporter 1; 18F-SynVesT-1: 18 fluoride—Synaptic vesicle tracer 1; BA-F8: myosin heavy chain type I antibody clone BA-F8; SC-71: myosin heavy chain type IIa antibody clone SC-71; BF-F3: myosin heavy chain type IIb antibody clone BF-F3; 6HI: myosin heavy chain type IIx antibody clone 6HI; GAD67: glutamate decarboxylase 67; TH: tirosina-hidroxilase; LSCI: laser speckle contrast imaging; ELISA: enzyme-linked immunosorbent assay; RT-qPCR: reverse transcription quantitative real-time polymerase chain reaction; LDF: Laser Doppler Flowmetry; NADPH: nicotinamide adenine dinucleotide phosphate; TEM: transmission electron microscopy; NO: nitric oxide; NOS: nitric oxide synthase; qPCR: quantitative real-time polymerase chain reaction; rs-fMRI: resting-state functional magnetic resonance imaging; CS: cortical stimulation; PBMT: photobiomodulation therapy; AR: androgen receptor; BBB: blood–brain barrier; NPCs: neural progenitor cells; STN: subthalamic nucleus; HFS: high-frequency stimulation; LFP: local field potential; BMI: brain machine interfaces; GABAAR: γ-aminobutyric acid type A receptor; AMPK: 5′-AMP-activated protein kinase; mTOR: mechanistic target of rapamycin; Bax: Bcl-2-associated protein X; Bcl-2: B-cell lymphoma protein 2; rNPFF: recombinant neuropeptide FF; ROS: reactive oxygen species; SOD: superoxide dismutase; GSH: glutathione; PC12: pheochromocytoma 12 cell line; OGD: oxygen-glucose deprivation; mGluR5: type-5 metabotropic glutamate receptor; NAM: negative allosteric modulators; PLGA: poly lactic-co-glycolic acid; DA: dopamine; CST: corticospinal tract; WM: white matter; p-AMPK: phosphorylated 5′-AMP-activated protein kinase; P70-S6K: 70-kDa ribosomal protein S6 kinase 1; w: week; h: hours; d: days.

## Data Availability

The original contributions presented in the study are included in the article. Further inquiries can be directed to the corresponding author.
